# Signatures of Adaptation and Purifying Selection in Highland Populations of *Dasiphora fruticosa*

**DOI:** 10.1093/molbev/msae099

**Published:** 2024-05-20

**Authors:** Fu-Sheng Yang, Min Liu, Xing Guo, Chao Xu, Juan Jiang, Weixue Mu, Dongming Fang, Yong-Chao Xu, Fu-Min Zhang, Ying-Hui Wang, Ting Yang, Hongyun Chen, Sunil Kumar Sahu, Ruirui Li, Guanlong Wang, Qiang Wang, Xun Xu, Song Ge, Huan Liu, Ya-Long Guo

**Affiliations:** State Key Laboratory of Systematic and Evolutionary Botany, Institute of Botany, Chinese Academy of Sciences, Beijing 100093, China; China National Botanical Garden, Beijing 100093, China; College of Life Sciences, University of Chinese Academy of Sciences, Beijing 100049, China; State Key Laboratory of Agricultural Genomics, Key Laboratory of Genomics, Ministry of Agriculture, BGI Research, Shenzhen 518083, China; BGI Research, Wuhan 430074, China; State Key Laboratory of Agricultural Genomics, Key Laboratory of Genomics, Ministry of Agriculture, BGI Research, Shenzhen 518083, China; BGI Research, Wuhan 430074, China; State Key Laboratory of Systematic and Evolutionary Botany, Institute of Botany, Chinese Academy of Sciences, Beijing 100093, China; China National Botanical Garden, Beijing 100093, China; State Key Laboratory of Systematic and Evolutionary Botany, Institute of Botany, Chinese Academy of Sciences, Beijing 100093, China; China National Botanical Garden, Beijing 100093, China; College of Life Sciences, University of Chinese Academy of Sciences, Beijing 100049, China; State Key Laboratory of Agricultural Genomics, Key Laboratory of Genomics, Ministry of Agriculture, BGI Research, Shenzhen 518083, China; State Key Laboratory of Agricultural Genomics, Key Laboratory of Genomics, Ministry of Agriculture, BGI Research, Shenzhen 518083, China; State Key Laboratory of Systematic and Evolutionary Botany, Institute of Botany, Chinese Academy of Sciences, Beijing 100093, China; China National Botanical Garden, Beijing 100093, China; State Key Laboratory of Systematic and Evolutionary Botany, Institute of Botany, Chinese Academy of Sciences, Beijing 100093, China; China National Botanical Garden, Beijing 100093, China; College of Life Sciences, University of Chinese Academy of Sciences, Beijing 100049, China; State Key Laboratory of Systematic and Evolutionary Botany, Institute of Botany, Chinese Academy of Sciences, Beijing 100093, China; China National Botanical Garden, Beijing 100093, China; College of Life Sciences, University of Chinese Academy of Sciences, Beijing 100049, China; State Key Laboratory of Agricultural Genomics, Key Laboratory of Genomics, Ministry of Agriculture, BGI Research, Shenzhen 518083, China; State Key Laboratory of Agricultural Genomics, Key Laboratory of Genomics, Ministry of Agriculture, BGI Research, Shenzhen 518083, China; State Key Laboratory of Agricultural Genomics, Key Laboratory of Genomics, Ministry of Agriculture, BGI Research, Shenzhen 518083, China; BGI Research, Wuhan 430074, China; College of Life Sciences, University of Chinese Academy of Sciences, Beijing 100049, China; State Key Laboratory of Agricultural Genomics, Key Laboratory of Genomics, Ministry of Agriculture, BGI Research, Shenzhen 518083, China; State Key Laboratory of Agricultural Genomics, Key Laboratory of Genomics, Ministry of Agriculture, BGI Research, Shenzhen 518083, China; State Key Laboratory of Systematic and Evolutionary Botany, Institute of Botany, Chinese Academy of Sciences, Beijing 100093, China; China National Botanical Garden, Beijing 100093, China; College of Life Sciences, University of Chinese Academy of Sciences, Beijing 100049, China; State Key Laboratory of Agricultural Genomics, Key Laboratory of Genomics, Ministry of Agriculture, BGI Research, Shenzhen 518083, China; State Key Laboratory of Systematic and Evolutionary Botany, Institute of Botany, Chinese Academy of Sciences, Beijing 100093, China; China National Botanical Garden, Beijing 100093, China; College of Life Sciences, University of Chinese Academy of Sciences, Beijing 100049, China; College of Life Sciences, University of Chinese Academy of Sciences, Beijing 100049, China; State Key Laboratory of Agricultural Genomics, Key Laboratory of Genomics, Ministry of Agriculture, BGI Research, Shenzhen 518083, China; State Key Laboratory of Systematic and Evolutionary Botany, Institute of Botany, Chinese Academy of Sciences, Beijing 100093, China; China National Botanical Garden, Beijing 100093, China; College of Life Sciences, University of Chinese Academy of Sciences, Beijing 100049, China

**Keywords:** adaptation, conservation biology, comparative genomics, *Dasiphora fruticosa*, genetic load, population genomics, purifying selection, highland

## Abstract

High mountains harbor a considerable proportion of biodiversity, but we know little about how diverse plants adapt to the harsh environment. Here we finished a high-quality genome assembly for *Dasiphora fruticosa*, an ecologically important plant distributed in the Qinghai-Tibetan Plateau and lowland of the Northern Hemisphere, and resequenced 592 natural individuals to address how this horticulture plant adapts to highland. Demographic analysis revealed *D. fruticosa* underwent a bottleneck after Naynayxungla Glaciation. Selective sweep analysis of two pairs of lowland and highland populations identified 63 shared genes related to cell wall organization or biogenesis, cellular component organization, and dwarfism, suggesting parallel adaptation to highland habitats. Most importantly, we found that stronger purging of estimated genetic load due to inbreeding in highland populations apparently contributed to their adaptation to the highest mountain. Our results revealed how plants could tolerate the extreme plateau, which could provide potential insights for species conservation and crop breeding.

## Introduction

Mountains are disproportionately important for Earth's biodiversity, and areas with high biodiversity are often associated with topographic uplift, which leads to environmental changes (Antonelli et al. 2018; Chen et al. 2019; Rahbek et al. 2019a, 2019b; Igea and Tanentzap 2021; Van Belleghem et al. 2023). Global mountain building has triggered the radiation of alpine species and elevated speciation (Ding et al. 2020; Wang et al. 2021; Lou et al. 2022). The Qinghai-Tibetan Plateau (QTP) is the largest and highest plateau in the world, which harbors numerous endemic alpine species (Zhang et al. 2019b; Zeng et al. 2020). However, the impact of the formation of QTP in creating high biodiversity remains poorly understood (Ma et al. 2014; Hu et al. 2022; Xia et al. 2022). It is hypothesized that altitude imparts a syndrome of developmental and physiological characteristics (including dwarfism, low-temperature tolerance, resistance to ultra-violet (UV) light, and changes in flowering time) linked mostly to climatic adaptation (Abbas et al. 2022). Therefore, understanding how different plant species adapt to the harsh alpine environment, characterized by cold, drought, and strong UV-light, is an intriguing question, particularly in the context of rapid climate change. It is crucial to investigate how plants cope with extreme conditions to disentangle the influence of abiotic or biotic stresses that significantly affect the growth and development of crops (Guo et al. 2020; Zeng et al. 2020; Li et al. 2021).

**Fig. 1.**
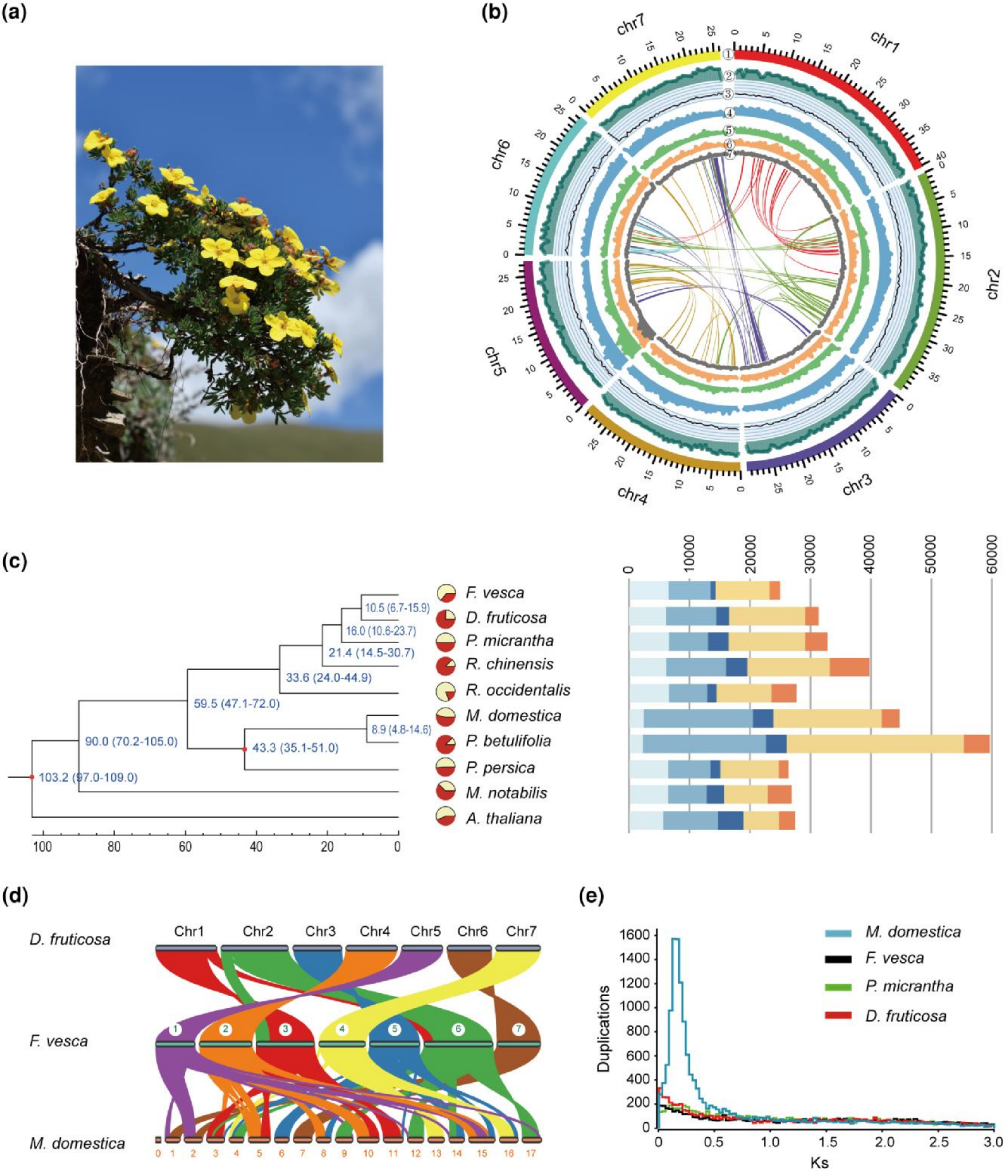
Assembly, annotation and comparative genomics of the *D. fruticosa* genome. a) Habitat and flowers were photographed (by Ze Wei) in the wild population. b) Synteny and distribution of genomic features. **①** Pseudochromosomes; **②** Gene number density; **③** GC density; **④** Repeat density; **⑤** LTR density; **⑥** Copia density; **⑦** Gypsy density. c) Expansion and contraction of gene families in *D. fruticosa* and the nine reference species (*Fragaria vesca, Potentilla micrantha, Malus domestica, Prunus persica, Pyrus betulifolia, Rosa chinensis, Rubus occidentalis, Morus notabilis, Arabidopsis thaliana*), with the dark red pie chart showing the expansion and the light blue showing the contraction. The right panel shows the proportions of orthogroups, with the light blue, medium blue, and blue showing single copy orthologs, multiple copy orthologs, and unique paralogs, respectively; and the pale orange and orange showing other orthologs and unclustered genes, respectively. d) Synteny between *D. fruticosa*, *F. vesca* and *M. domestica*. e) *Ks* plot of *D. fruticosa*, *F. vesca*, *M. domestica,* and *P. micrantha.*

*Dasiphora* is a genus of shrubs in the rose family (Rosaceae) that are native to southwestern China and other regions of the Northern Hemisphere. It has been used as a landscape ornamental plant since the early 17th century (Davidson and Lenz 1989). These shrubs are known as “Galsang flowers” by Tibetan people and are believed to bring happiness and luck. They can grow at an altitude of up to 5,500 m and can survive temperatures as low as −50 °C. The appearance of *Dasiphora* shrubs varies depending on their altitude. At low altitudes, they are erect, while at high altitudes, they are prostrate. Species of *Dasiphora* were previously treated as members of the genus *Potentilla*. However, a recent molecular phylogenetic study indicated that *Dasiphora* is more closely related to *Fragaria* than to *Potentilla* (Dobeš and Paule 2010). The *Dasiphora* genus includes five species and numerous hybrids (Davidson and Lenz 1989). There are two species distributed in China (Ma et al. 2014): the yellow-petaled *D. fruticosa* and the white-petaled *D. glabra*. *Dasiphora fruticosa* (Fig. 1a) is a widespread species in the QTP and its surrounding mountains. This species is characterized by pinnately compound leaves and five-petaled yellow flowers, while the number, shape, and size of the leaflets vary, as does the pubescence on the leaves. It can be found in a variety of habitats, including hills, mountains, and valleys. A few populations have also been found at lower altitudes in northern China. Phylogeographic studies of *D. fruticosa* based on cpDNA fragments detected radical population expansions across the whole QTP before the last glacial maximum (Ma et al. 2014). It emerges as a great model to understand adaptation to the highest plateau.

Here we sequenced *D. fruticosa* genome using multiple sequencing strategies, including Oxford Nanopore long reads, BGI short reads and Hi-C paired-end reads, and resequenced 592 natural accessions. Comparative and population genomics revealed the evolutionary history of *D. fruticosa,* and uncovered its adaptive mechanisms to the highest plateau. In particular, we found that stronger purging of estimated genetic load in highland populations contributes to its adaptation. Our results shed light on the plant adaptation to the extreme environment and our understanding of the formation of global mountain biodiversity.

## Results

### Assembly and Annotation of *D. fruticosa* Genome Sequence

We generated a total of 54 Gb (∼ 245×) raw Oxford Nanopore long-read, of which 47 GB (∼214×) passed QC were assembled into contigs and subsequently polished using 67 Gb (∼308×) BGI-seq short reads (supplementary table S1, Supplementary Material online). The final assembled genome is 222.5 Mb (coverage 91.5%) with a contig N50 length of 2.1 Mb, which is similar to the estimated size of 243 Mb based on *k*-mer analysis (Table 1; supplementary table S2 and fig. S1, Supplementary Material online). A total of 100 Gb (∼454×) Hi-C paired-end reads were mapped to the contig assembly, with a scaffold N50 length of 29.39 Mb. In total, 98.69% contigs were anchored to the 7 pseudochromosomes (222.63 Mb in length) (Fig. 1b; supplementary fig. S2; tables S3 and S4, Supplementary Material online), consistent with the reported chromosome number (2n = 14). BUSCO analysis suggested that 97.5% of complete embryophyte-conserved genes could be identified in the *D. fruticosa* genome (Table 1; supplementary table S5, Supplementary Material online). Moreover, the mapping rate of short reads to the assembled scaffold genome is 97.3% (Table 1; supplementary table S6, Supplementary Material online), and the LTR Assembly Index (LAI) is 12.6 (Table 1). All these results indicated that the assembled *D. fruticosa* genome is a high-quality genome in terms of contiguity and completeness.

**Table 1:** Assembly and annotation statistics of *D. fruticosa* genome

Genome characteristics	*D. fruticosa*
Assembly	
Estimated genome size	243.57 Mb
Assembled genome size	222.57 Mb
GC content	38.99%
N50 of contigs (bp)	2,106,498
N50 of scaffolds (bp)	3,535,763
Total length of contig (bp)	222,571,321
Total length of scaffold (bp)	222,573,099
Longest scaffold	15,857,080
Complete BUSCOs	97.50%
LAI (LTR Assembly Index)	12.58
Genome annotation	
Repeat region	41.09%
Number of protein-coding genes	31,351
Average length of transcripts (bp)	2,887.92
Average exon length (bp)	236.05
Average intron length (bp)	384.22
Hi-C	
Anchor size	219.68
Anchor rate	98.70%
Number of pseudochromosomes	7
N50 of scaffold (bp)	27,726,264
Longest scaffold (bp)	40,277,584

A total of 20 Gb of clean RNA-seq reads were used to annotate the protein-coding genes (supplementary table S1, Supplementary Material online). Combining de novo, homologue-based and transcription-based algorithms, we predicted 31,351 protein-coding genes in the *D. fruticosa*, and more than 24,858 of *Fragaria vesca* (Table 1; supplementary table S7, Supplementary Material online). In the *D. fruticosa* genome, the complete BUSCO of protein-coding genes was 93.9% (supplementary table S8, Supplementary Material online). More than 93.7% of the genes were assigned to a suite of function databases (supplementary table S9, Supplementary Material online). There were 1,450 transcription factor genes, which could be classified into 56 different families (supplementary table S10, Supplementary Material online). The annotation of noncoding RNAs (ncRNAs) in *D. fruticosa* identified 1,741 ncRNAs, including 690 ribosomal RNAs (rRNAs), 543 transfer RNAs (tRNAs), 103 microRNA, and 405 small nuclear RNAs (snRNAs), and in particular, the number of 5S rRNAs in *D. fruticosa* was higher than that in *F. vesca* (supplementary table S11, Supplementary Material online).

Transposable elements usually take up a large fraction of plant genomes. The genome of *D. fruticosa* contained 41.1% transposable elements (TEs) (supplementary table S12, Supplementary Material online), higher than that in *F. vesca* (∼35.7%) (Shulaev et al. 2011). In particular, there were ∼5.5% LINE in the *D. fruticosa* genome, much higher than that of *F. vesca* genome (∼1.9%) (supplementary table S12, Supplementary Material online).

### Gene Family, Phylogenetic, and Whole-genome Duplication Analyses

To identify the gene families of *D. fruticosa* and nine representative species (*F. vesca*, *P. micrantha*, *M. domestica*, *Prunus persica*, *Pyrus betulifolia*, *Rosa chinensis*, *Rubus occidentalis*, *Morus notabilis*, and *Arabidopsis thaliana*), OrthoFinder was used for orthologue identification. In total, we identified 27,441 gene families in these 10 species, and 677 species-specific gene families in the *D. fruticosa* genome (Fig. 1c, supplementary table S13, Supplementary Material online). A total of 592 single-copy orthologues were identified and used to construct a phylogenetic tree of 10 representative species. Both the concatenated and coalescent-based trees indicated that *D. fruticosa* and *F. vesca* formed a sister clade to *Potentilla micrantha* (Fig. 1c) and diverged from the latter about 16.0 million years ago (MYA) (supplementary fig. S3, Supplementary Material online). The divergence time between *D. fruticosa* and *F. vesca* was 10.5 MYA (supplementary fig. S3, Supplementary Material online). To investigate potential whole-genome duplication (WGD) events, the number of synonymous substitutions per synonymous site (*Ks*) for all syntenic paralogue/orthologue pairs was calculated for *D. fruticosa*, *F. vesca*, *P. micrantha*, and *Malus domestica*, respectively. Our results showed that only *M. domestica* has experienced a recent WGD event (Su et al. 2021), but not in the other three species (Fig. 1d and e).

The GO enrichment showed that these 677 species-specific gene families in *D. fruticosa* were enriched in 65 GO terms (p < 0.05) of biological process (BP), including the lycopene biosynthetic process (GO:1901177), cellular response to DNA damage stimulus (GO:0006974), DNA repair (GO:0006281), and carotenoid biosynthetic process (GO:0016117) (supplementary table S14, Supplementary Material online). Among these biological processes, carotenoid biosynthetic process plays a vital role in either photoprotection or light-harvesting (Cazzonelli and Pogson 2010; Vranová et al. 2013). The carotenoids include two major groups: carotenes (mainly β-carotene) enriched in the photosystem reaction centers, and xanthophylls abundant in the light-harvesting complexes, as molecules required for protecting photosynthetic organisms from the potentially toxic effects of light (De Souza et al. 2022).

Based on our assembled genome, we reconstructed the entire metabolic pathway for carotenoid biosynthesis in *D. fruticosa* and identified 30 genes (supplementary fig. S4A and table S15, Supplementary Material online) encoding enzymes in the pathway. Many genes of the pathway expanded in the *D. fruticosa* genome in comparison to *F. vesca*: *PDS* (3:1), *ZDS* (7:1), *LCYB* (2:1), *ZEP* (4:1) (supplementary fig. S4B and table S15, Supplementary Material online), and the former is characterized by yellow to orange-yellow flower while the latter is white-flowered. The yellow color of flower might play a vital role in photoprotection (Cazzonelli and Pogson 2010; Vranová et al. 2013). It has been demonstrated that *PDS* may play a rate-limiting role in the generation of carotene (Cazzonelli and Pogson 2010), and ZEP is the key enzyme of the xanthophyll cycle, playing important roles in plant photoprotection and serving as precursors for the plant hormone abscisic acid (North et al. 2005). Paralogs of these enzymatic genes of the pathway may facilitate adaptation in dynamic environments, which is particularly important for plants (Vranová et al. 2013). Therefore, the expansion of these enzymatic genes may have increased the contents of carotene, lutein, and zeaxanthin in *D. fruticosa*, facilitating plants to improve the ability of UV photoprotection and cold tolerance.

The rapidly expanded and contracted gene families might be correlated with the adaptation to the high-altitude environment. There were 256 gene families that comprise 2,347 expanded genes (p < 0.05), and 82 gene families that comprise 246 contracted genes (p < 0.05) in the *D. fruticosa* (Fig. 1c; supplementary fig. S5, Supplementary Material online). The GO enrichment of those genes was mainly enriched in signal transduction (BP, GO:0007165), cellular response to stimulus (BP, GO:0051716), response to abiotic stimulus (BP, GO:0009628), regulation of cellular process (BP, GO:0050794), regulation of BP (GO:0050789) (supplementary table S16, Supplementary Material online).

### Phylogeographic and Population Structure Analyses Clarified its Demographic History

**Fig. 2.**
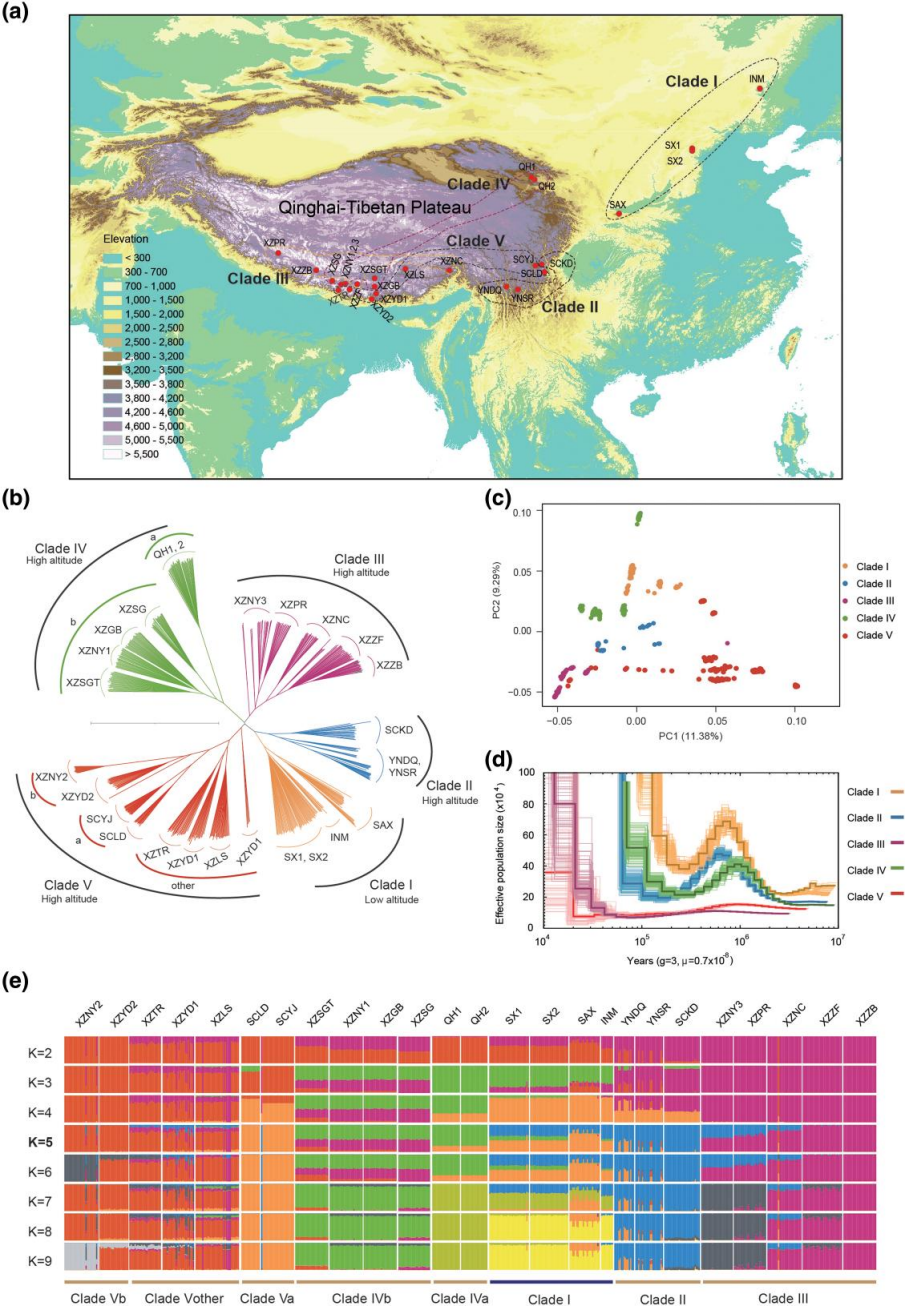
Demographic history analyses of 25 populations of *D. fruticosa*. a) Geographical locations of sample collection site for resequencing. b) Neighbor-joining phylogenetic analysis. c) PCA analysis. d) Population–demographic history inferred with PSMC. Different colors represent different clades. e) Population structure based on admixture analysis.

A total of 599 accessions of *D. fruticosa* from 21 highland populations and 4 lowland populations were sequenced, and 592 quality accessions were analyzed in this study (Fig. 2a; supplementary table S17 and file S1, Supplementary Material online). We obtained 65.78 Gb high-quality clean reads with an average 41.1× coverage per accession, which amounts to 24,328.1× coverage in total (supplementary file S1, Supplementary Material online). The mapping rate of accessions to the genome of *D. fruticosa* varied from 70.9% to 94.2% (average 84.4%) (supplementary file S1, Supplementary Material online). In total, we identified 84,530,065 SNPs and 31,407,195 small indels, after filtering the missing rate and minor allele frequency (MAF < 0.05) of SNPs, a set of 2,956,203 high-quality single-nucleotide polymorphisms (SNPs) were kept for further analysis (supplementary table S18, Supplementary Material online).

To explore the genetic relationship among samples, phylogenetic analysis, principal component analysis (PCA), and admixture analysis were performed based on 1,857,214 nonlinkage SNPs (Fig. 2). There are five clades based on phylogenetic analysis (Fig. 2b). All individuals from low latitudes (populations of Inner Mongolia, Shanxi, and Shaanxi) formed a monophyletic clade I (INM, SX1, SX2, SAX). The QTP populations were divided into four clades (II, III, IV, V). Clade II comprised two populations (YNDQ, YNSR) from Yunnan and one population (SCKD) from Kangding, Sichuan province, and these three populations inhabit the southeastern region of the QTP. Xizang populations were clustered into three clades (III, IV, V). Populations of the clade III were found to be mainly distributed in the central and western Himalayas. The clade IV was divided into two subclades (IVa, IVb), the former (population QH1, QH2) is distributed in the northeastern part (Qinghai province) of QTP and the later (population XZSG, XZGB, XZNY1, XZSGT) are distributed around the Qomolangma Nature Reserve. In the clade V, Va (SCLD, SCYJ) in the eastern region (Sichuan province) of the QTP and Vb (XZYD2, XZNY2) distributed in the central Himalaya formed sister clades. In addition, the results of PCA and ADMIXTURE (K = 5) are largely consistent with that of the phylogenetic analysis (Fig. 2c and e).

To characterize each local population, we compared the nucleotide diversity (π) (supplementary table S19, Supplementary Material online) between the high-altitude population group (clades II, III, IV, V) and the low-altitude population group (clade I) in a window size of 50 kb and step size of 10 kb. The π values of the high-altitude group (median = 3.1 × 10^−3^) were significantly higher than those of the low-altitude group (median = 2.9 × 10^−3^) (p < 0.001). In particular, the π values (median = 3.5 × 10^−3^) of the clade IVb distributed in Xizang were significantly higher than those (median = 2.9 × 10^−3^) of the clade IVa in the northeastern edge of the QTP (p < 0.001) (supplementary table S19, Supplementary Material online), and the π values of the clade Vb (median = 1.7 × 10^−3^) distributed in the central Himalaya were significantly higher than those of the clade Va in the eastern edge of the QTP (median = 1.3 × 10^−3^) (p < 0.001). The demographic history analysis using PSMC showed that *D. fruticosa* populations underwent a bottleneck of about 0.5 MYA (Fig. 2d) after the Naynayxungla Glaciation when large ice caps were covering a total area ⩾500,000 km^2^ in the QTP.

### Interspecific and Intraspecific Analyses About the Adaptation to the Highest Mountain

To identify candidate genes for high-altitude adaptations, we performed the genome scan at both interspecific and intraspecific analyses. At the interspecific level, we calculated the *ka/ks* value between *D. fruticosa* and its related species *F. vesca*, and detected 113 genes with *ka/ks* values above 1. These genes might have contributed to the adaptation to the highest mountain. The GO enrichment showed these genes were enriched in negative regulation of cellular process (BP, GO:0048523, *P* < 0.05), negative regulation of phosphoprotein phosphatase activity (BP, GO:0032515), mature ribosome assembly (BP, GO:0042256), response to stimulus (BP, GO:0050896), and DNA damage checkpoint (BP, GO:0000077) (supplementary table S20, Supplementary Material online).

At the intraspecific level, we conducted the genome scan to identify genes under selection using two strategies, two-population comparison method between high- and low-altitude populations, and single-population method in the high-altitude populations. For the first strategy, two analyses (*F*_ST_ and XP-CLR) under a 50 kb window with a 10 kb step size (5% cutoff) (supplementary table S21, Supplementary Material online) were performed, and genes showed up in both methods were regarded as candidate genes under selection. The identified genes under selection might be correlated with the adaptation to the high-altitude environment.

**Fig. 3.**
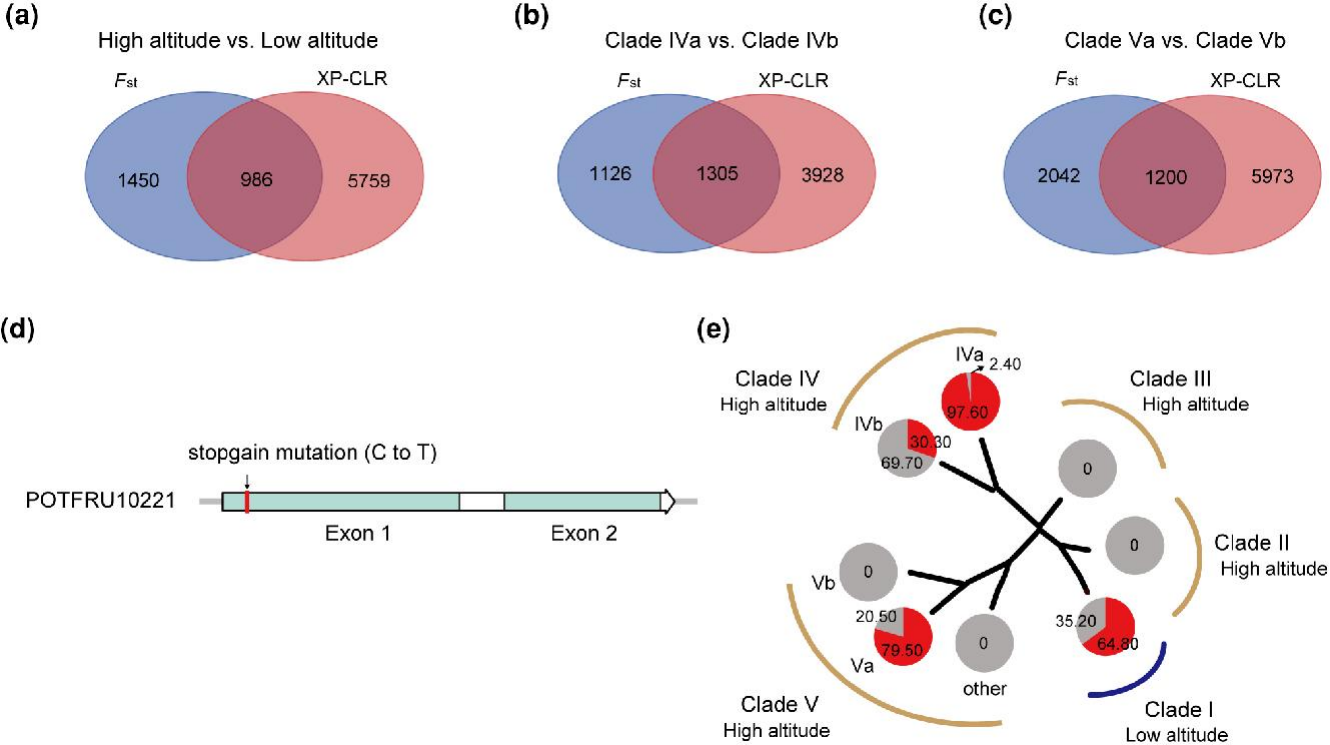
Genome-wide distribution of selective sweeps in the high-altitude populations of *D. fruticosa*. a) The intersection of genes under selection based on *F*_ST_ and XP-CLR between the high-altitude (clades II, III, IV, V) and low-altitude (clade I) populations. b) The intersection of genes under selection based on *F*_ST_ and XP-CLR between clade IVa and IVb. c) The intersection of genes under selection based on *F*_ST_ and XP-CLR between clade Va and Vb. d) The gene structure and the stopgain SNP site of *POTFRU10221*. e) The mutation frequency of the stopgain SNP sites of *POTFRU10221* in each clade. The pie showed the proportion of mutant individuals accounting for the total individuals in each clade, red means the proportion of mutant individuals, and gray means the proportion of individuals without mutation.

First, we scanned the genomic regions under selection in the high-altitude population group (clades II, III, IV, V) against the low-altitude population group (clade I), and obtained 986 candidate genes associated with high-altitude adaptation (Fig. 3a). These genes are enriched in 7 GO terms of BP, including the response to biotic stimulus (BP, GO:0009607) (supplementary file S2, Supplementary Material online). Among these outliers, it has been reported that *GAPDH*, *MDHs*, *CAR*, and *PRL1* are involved in plant development regulation and abiotic stress tolerance (Chernyad’ev and Monakhova 2006; Flores-Pérez et al. 2010; Rodriguez et al. 2014; Zhang et al. 2019a, 2022; Nan et al. 2020; Yang et al. 2020). *LRX*, *WIN2*, *LBD20*, *HS1* are associated with cell wall formation and pathogen defense (Lee et al. 2008; Thatcher et al. 2012). Moreover, *SODCC.2* and *FSD3* are the members of *SODs* that might involve in scavenging reactive oxygen species (ROS) accumulated under cold, drought, and UV-B stresses (supplementary file S3, Supplementary Material online).

Second, we scanned genes under selection in the two subclade pairs of clades IV and V to identify variants underlying adaptations in the two central QTP population groups [clade IVb (populations XZSG, XZGB, XZNY1, XZSGT), Vb (XZYD2, XZNY2)] against the two plateau-edge population groups [clade IVa (QH1, QH2), Va (SCLD, SCYJ)], respectively. We identified 1,305 (Fig. 3b) and 1,200 (Fig. 3c) selection candidate genes in the two high-altitude central groups, respectively. The 1,305 genes identified in clade IVb are enriched in 10 GO terms of BP, including the wax metabolic process (BP, GO:0010166), positive regulation of immune system process (BP, GO:0002684), response to external stimulus (BP, GO:0009605), and abiotic stimulus (BP, GO:0009628) (supplementary file S4, Supplementary Material online). Among these genes (supplementary files S4 and S5, Supplementary Material online), *ECR*, *LACS*, and *KCS* associated with wax biosynthesis are likely involved in protection against UV radiation and drought. *ABC1K8*, *SODs*, and *FSDs* are associated with an antioxidant defense under cold and UV stresses (Manara et al. 2016). *KIN17*, *RAD4,* and *RAD51* are functionally correlated to DNA replication and DNA repairs (Masson et al. 2003; Gong et al. 2006; Bonilla et al. 2020). Moreover, mutations of either *GA20ox1* or *GA3ox1* can cause dwarfing varieties in *A. thaliana* (Mitchum et al. 2006; Rieu et al. 2008). In contrast, the 1,200 high-altitude selection candidate genes identified in the clade Vb group are enriched in biosynthetic process (GO:0009058) and cell cycle checkpoint (GO:0000075) (supplementary file S6, Supplementary Material online). Among these genes, *GA20ox1* and *GA3ox1* show significant selection signals, and candidate genes involved in cell wall architecture (e.g. *CESA6*, *CSLG2*, *CAD9*, *4CL1*) and carotenoid biosynthesis (e.g. *ZDS*, *NCED1*) were identified (supplementary file S7, Supplementary Material online).

There are 63 high-altitude selection candidate genes shared in the two subclade pairs of clade IV and V (supplementary fig. S6A, Supplementary Material online), which suggest that the high-altitude plants on the plateau have experienced parallel evolution. These genes are mainly enriched in 11 GO terms of BP, including the regulation of molecular function (GO:0065009) and cell wall organization or biogenesis (GO:0071554), which are important for plants adapting to the highest mountain (supplementary file S8, Supplementary Material online). Interestingly, three *PME* (pectin methylesterase) genes (*POTFRU10220*, *POTFRU10221*, *POTFRU10222*) significantly enriched on the regulation of molecular function, which are present in tandem duplication in the *D. fruticosa* genome (supplementary fig. S6, B-D, Supplementary Material online). Intriguingly, we found a stopgain SNP site (Fig. 3d) in the gene *POTFRU10221* that appears at substantially lower frequencies in the high-altitude population groups (clades II-V, IVb, Vb), respectively (Fig. 3e; supplementary fig. S6, B-D, and file S9, Supplementary Material online). Phylogenetic analysis of the *PME* gene family indicated that *POTFRU10221* belongs to the homolog member of the *AT2G26440* (*PME12*) in *A. thaliana* (supplementary fig. S6C, Supplementary Material online). Moreover, *GA20ox1* and *GA3ox1* that are the key oxidase enzymes for the bioactive gibberellin biosynthesis are also under selection in the populations of two subclades (IVb and Vb), implying dwarfism is an important strategy to adapt to the extreme environment of the highest mountain.

For the second strategy, we identified 219 regions (5% cutoff) containing 237 genes under selective sweeps in the high-altitude population group using SweeD software. There were 13 genes overlapped with the 986 genes identified from the intersection of *F*_ST_ and XP-CLR with the first strategy, which include *SEP1* (Stress enhanced protein 1), *NHD1* (Sodium/proton antiporter), *NDUA5* (NADH dehydrogenase), and F-box proteins and *WRKY27* (WRKY transcription factor 27) (supplementary file S10, Supplementary Material online).

### Deleterious Mutation Accumulation Among Different Clades

**Fig. 4.**
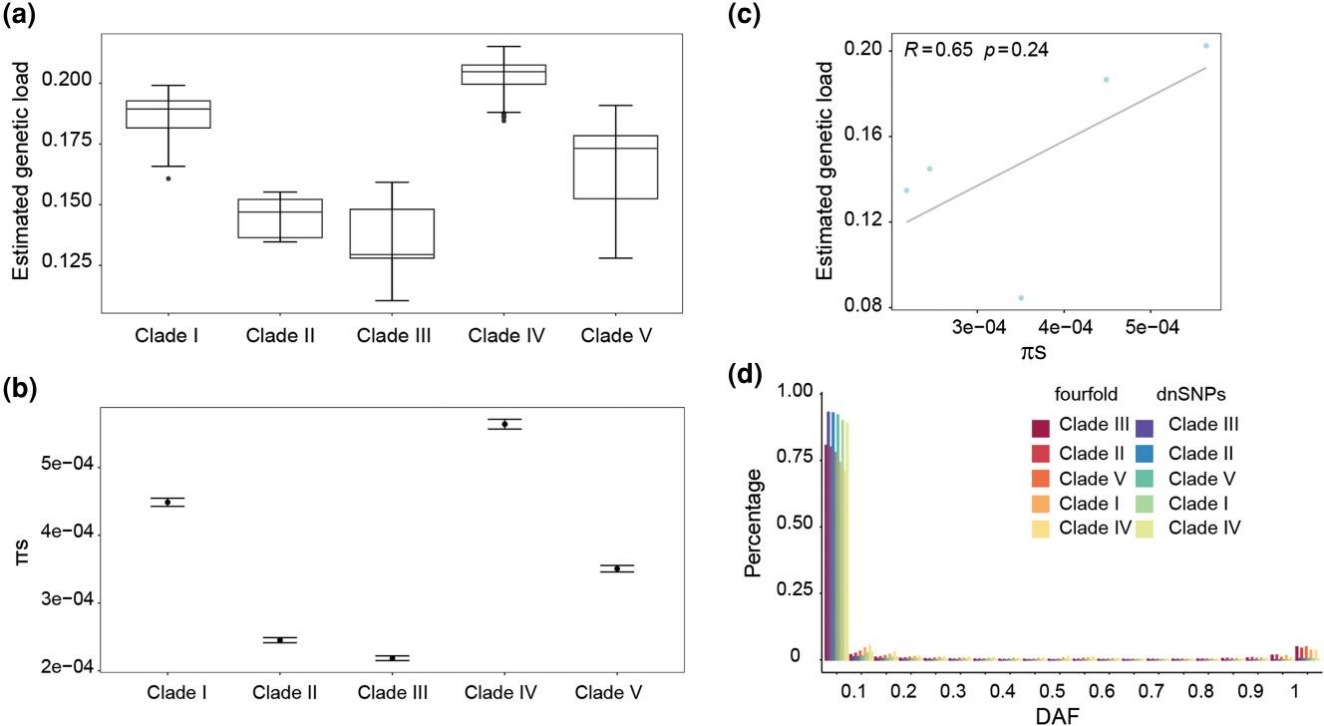
Deleterious mutation accumulation in different populations. a) The estimated genetic load in different clades. b) Fourfold degenerate sites diversity (πs) in different clades. Genetic diversity was calculated in nonoverlapping 10 kb windows. c) The correlation between effective population size (πs) and estimated genetic load. d) The site frequency spectrum (SFS) of 4-fold degenerate sites and deleterious nonsynonymous mutations (dnSNPs) in different clades. DAF, derived allele frequency.

To estimate the accumulation of deleterious mutations in different clades, we identified deleterious nonsynonymous mutations (dnSNPs) using SIFT 4G (Vaser et al. 2016). Using *F. vesca* and *Rosa chinensis* as outgroups, we counted the ratio of derived allele number of dnSNPs to that of 4-fold degenerate sites for each individual, which was used as a genetic load proxy (hereafter referred to as estimated genetic load). Compared to low-latitude populations (clade I), high-altitude populations (clades II, III, V) on average have accumulated less deleterious mutations (lower estimated genetic load), except for clade IV, which appear to have accumulated the highest estimated genetic load, even higher than low-altitude populations (Fig. 4a). The higher estimated genetic load of clade IV mostly probably resulted from the lower altitude of clade IVa than other high-altitude populations, and additionally, the highland clade IVb most probably derived from clade IVa.

In order to explore which factors contribute to the estimated genetic load differentiation among clades, we calculated the 4-fold degenerate site diversity (πs), which could measure the long-term effective population size (Lynch and Conery 2003). Consistent with the inference of population history (Fig. 2d), the low-altitude populations have a much larger population size than high-altitude populations (Fig. 4b and c). Among high-altitude populations, clade II, clade III, and clade V have a much smaller population size (Fig. 4b). In contrast to normally expected that small population has more deleterious mutation due to weak purifying selection (Robinson et al. 2023), here we found small populations appear to have accumulated lower estimated genetic load (Fig. 4a–c). The reduced estimated genetic load in highland populations with smaller effective population size are likely caused by enhanced purifying selection against recessive deleterious variants due to excess homozygosity induced by inbreeding (or drift) in small populations caused by fragmentation, known as purging (Dussex et al. 2023).

To validate the assumption of strong purifying selection against recessive deleterious variants, we estimated the site frequency spectrum (SFS) of 4-fold degenerate sites and dnSNPs, and found that high-altitude populations (clades II, III, V) have an excess of rare dnSNPs than that in low-latitude populations (clade I) (Fig. 4d), supporting our assumption. The estimated SFS of 4-fold degenerate sites and dnSNPs exhibit a similar trend of the loss of common variants, and the SFS of the former shows a sign of a U-shaped distribution (Fig. 4d), implying that linked selection and/or migration have affected more of the genomic regions than is encompassed within the identified selective regions, similar to the scenes in other studies (Caicedo et al. 2007; Liu et al. 2017). To test if inbreeding contributed to the variation of estimated genetic load, we performed runs of homozygosity (ROH) analysis in each clade, and found that high-altitude populations have a larger total number of longer ROH than low-latitude populations (supplementary fig. S7 and table S23, Supplementary Material online), which support the recent inbreeding induced by population bottleneck. Taken together, our results suggest that the highland populations have accumulated proportionally fewer nonsynonymous mutations via genetic purging.

## Discussion

To understand the mystery of how alpine species adapt to high mountain, we generated a high-quality, chromosome-scale genome assembly for an ecologically important alpine species *D. fruticosa* and performed resequencing for 592 natural accessions, which also provide important genetic resources for developing novel cultivars.

Candidate genes associated with high-altitude adaptation were identified by genome-wide analyses of gene families, interspecific and intraspecific analyses. We detected 677 species-specific gene families in *D. fruticosa*, likely involve in the lycopene biosynthetic process, cellular response to DNA damage stimulus, and DNA repair. In particular, selection signals uniquely present in the high-altitude populations may be associated with cell wall formation, wax biosynthesis and/or antioxidant defense, which might be either involved in the response to cold-stress and UV radiation, and/or in scavenging ROS accumulated under cold and UV-light stresses. Furthermore, we found genes under positive selection in two high-altitude clades (IVb, Vb) of the main clades (IV, V), demonstrating parallel evolution for adaptation to the harsh environment in the QTP. Among these genes, *POTFRU10221*, a *PME* family member playing a large role in the function of the cell wall, has lower frequencies of stopgain in the high-altitude population groups (Fig. 3e), revealing that *D. fruticosa* probably adapts to the highest plateau through controlling cell wall architecture. The mechanical strength of the wall is vital for a plant to tolerate freezing stress and resist dehydration stress, and for a crop such as strawberry to increase yield. In addition, for alpine plants, the dwarfing trait is a common strategy to adapt to the harsh high-altitude environment. Two genes (*GA20ox1*, *GA3ox1*) associated with dwarfism in *Arabidopsis*, showed significant selection signals in the two parallel population groups (clades IVb, Vb), suggesting these two genes are associated with the adaptation to the harsh environment frequently. Therefore, these results reveal that the high-altitude populations have experienced parallel evolution, in which a specific set of genes involved in multiple physiological processes are under positive selection in both events.

Most importantly, we found that stronger purifying selection, due to inbreeding, removes deleterious mutation from highland populations and thus contributes to its adaptation. The reduced estimated genetic load in highland populations with smaller effective population size is caused by purifying selection against deleterious variants exposed in the homozygous state of small fragmented populations (Dussex et al. 2023). Taken together, these evidences suggest that *D. fruticosa* has adapted to high-altitude environments through multiple physiological processes, including dwarfism, tolerance to cold or UV radiation, and tolerance to other biotic or abiotic stresses, and particularly stronger purifying selection due to the recent inbreeding, induced by population bottleneck that prevents accumulation of expansion load in high-altitude populations, contributes to its adaptation to highland.

In this study, the comparative genomics and population genomics analyses revealed how the high-altitude populations of *D. fruticosa* adapted to the extreme environment of the highest plateau. Diverse climatic and topographic environments in the QTP (Favre et al. 2015; Antonelli et al. 2018; Rahbek et al. 2019a) may have rapidly promoted the genetic divergence of widely distributed species by shaping the connectivity and environmental conditions, and creating novel habitats and niches where new endemic lineages adapted. Overall, our study of the widely spread *D. fruticosa* revealed how an alpine species could adapt to the highest mountain, and provided the foundation and strategy for a better understanding of adaptation, and provided potential insights for species conservation and crop breeding.

## Materials and Methods

### Sample Preparation, Genome, and Transcriptome Sequencing

For whole-genome sequencing and assembly, we selected a *D. fruticosa* individual of Nyingchi population, located in Sejila Mountain, Xizang, China. High-quality DNA was extracted from fresh leaves by using QIAGEN Genomic kits, and the DNA quantification was checked by Nanodrop and Qubit. Nanopore GridION X5 sequencer was used for genome sequencing, with SQK_LSK108 Kit used to prepare the sequencing libraries (Cherf et al. 2012). For short read sequencing, BGI-DIPSEQ (Huang et al. 2017) was used for library constructing and sequencing. Finally, the DNA was digested by MboI according to the standard Hi-C library preparation protocol, then sequenced on BGI-DIPSEQ platform, generating ∼100 Gb Hi-C data (supplementary table S1, Supplementary Material online).

To support the gene annotation, a TIANGEN Kit was used for total RNA extraction from fresh leaves of the same individual for whole-genome sequencing. After quality control was performed using Agilent 2100, total RNA with RNA Integrity Number (RIN) around 7 was selected for library construction and sequencing on BGI-DIPSEQ platform.

The genome size of *D. fruticosa* was estimated based on BGI short reads through *k*-mer method using kmerfreq16bit (Version 2.4) (Marçais and Kingsford 2011). GCE (Liu et al. 2013) was used to estimate the heterozygosity and the repeat proportions (supplementary fig. S1, Supplementary Material online).

### *De Novo* Genome Assembly and Annotation

NECAT (Chen et al. 2021) was used for the nanopore long reads assembly of *D. fruticosa*, followed by a round of polish with the BGI short reads using NextPolish (Hu et al. 2019) with the default parameter. The assembled genome was reduced redundant by using purge_dups (v.1.2.3) (Guan et al. 2020) with the default settings.

The completeness and continuity of assembly were assessed by using BUSCO (V3.0.2) (Simão et al. 2015) software with embryophyta_odb10 (supplementary table S5, Supplementary Material online), then short reads were used by mapping to the genome by BWA (v.2.21). Finally, the RNA reads were mapped to the genome with Hisat2 (v. 2.1.0) (Kim et al. 2015) to evaluate the mapping rate (supplementary table S6, Supplementary Material online).

We combined de novo and homolog-based methods to identify the repeat element in the *D. fruticosa* genome. For de novo prediction, we used LTR_FINDER (Xu and Wang 2007), Piler, and repeatScout to detect different types of repeats and then built a nonredundant library to search the repeat element by RepeatMasker. For the homolog-based methods, we used TRF to detect tandem repeats, and RepeatMasker was used to search repeat element against with RepBase (v.21.12). LAI (LTR Assembly Index) was used to evaluate assembly continuity by evaluating the assembly of repeat sequences. First, LTRharvest (Ellinghaus et al. 2008) was used to detect LTR sequences with the parameter “-minlenltr 100 -maxlenltr 7000 -mintsd 4 -maxtsd 6 -motif TGCA -motifmis 1 -similar 85 -vic 10 -seed 20', then combined with the previous LTR_FINDER result. Finally, LTRretriever (v.2.8) was used to obtain high-confidence LTR retrotransposons and calculate the LAI score with the default settings (Ou et al. 2018).

The protein-coding genes were predicted by combining homology-based prediction, de novo prediction and RNA-seq-based prediction. GeneWise software (Madeira et al. 2022) was used for homology-based prediction against *Fragaria vesca* and *Potentilla micrantha* genomes. The result of Augustus hints from BRAKER2 (Brůna et al. 2021) was used for de novo prediction. For RNA-seq-based prediction, Hisat2 and stringtie were used to assemble the transcriptome, then TransDecoder (V2.0.1) was used for transcriptome mapping to the genome. Finally, EVidenceModeler (Haas et al. 2008) was used to combine the results from three prediction methods with the weights “ABINITIO_PREDICTION 4, PROTEIN 6, TRANSCRIPT 10'.

All protein-coding genes were blasted against NR, SwissProt, KOG, KEGG, and InterProScan, with an E-value cutoff of 1e-5 for functional annotation.

### Pseudochromosome Assembly by Using Hi-C

Juicer software (Durand et al. 2016) was used to extract the uniquely mapped and Hi-C contact reads without PCR duplication, then 3D-DNA (Dudchenko et al. 2017) was used to integrate the assembly genome into pseudochromosome-level assembly. Finally, the Hi-C assembly result was visualized by Juicebox and manually improved according to the Hi-C contact map.

### Orthogroup Inference and Phylogenetic Analysis

OrthoFinder (v 2.3.14) (Emms and Kelly 2015) combined BLAST and Markov Cluster Algorithm (MCL) program was used to identify orthogroups (OGs). First, the protein-coding sequences of each species were blasted with each other, using “all-vs-all” model and the parameters “blastp -outfmt 6 -evalue 0.001”; next, alignments were clustered into OGs using MCL program; finally, only single-copy OGs covering all species were retained for further analyses. For each single-copy OGs, we performed multiple amino acid sequence alignments using MAFFT (v.7.310) (Katoh and Standley 2013) and gap position removal using trimal (v1.4.1) (Capella-Gutiérrez et al. 2009).

We used concatenation and coalescent-based methods for phylogenetic analyses. For the concatenation method, all single-copy OGs were concatenated into a supermatrix to reconstruct a phylogenetic tree by using RAxML (v 8.2.4) with the parameters “-f a -#100 -m PROTCATJTT -p 12345 -x 12345'. For the coalescent-based method, each single-copy OGs was used for phylogenetic reconstruction by using RAxML (Stamatakis 2006) with the default settings. Then, all single-gene trees were inputted in the ASTRAL software to generate a coalescent-based species tree (Mirarab et al. 2014), with the parameters “-r 100 -m 0'. MCMCtree program (Yang 2007) was used for estimating divergence time with the parameters “–rootage 110 -type aa”. Café software (De Bie et al. 2006) was used to detect the expansion and contraction of gene families.

### Whole-Genome Duplication and Comparative Genome Analysis

*Ks* (synonymous substitutions per synonymous site) distribution was calculated using wgd program (Zwaenepoel and Van de Peer 2019) with the default parameters. The synteny between *D. fruticosa* and *F. vesca* was estimated using MCScanX (Wang et al. 2012). *Ka/Ks* values between colinear genes were estimated using the yn00 approach implemented in the PAML package (Yang 2007).

### Carotenoid Biosynthesis Pathway Analysis

The genes involved in the MEP pathway and carotenoid biosynthesis of *A. thaliana* were selected as query sequences, then BLASTP (NCBI Blast v 2.2.31) was used to align those query sequences against the protein-coding genes of each species (E-value 1e-5). Then the candidate genes were checked by functional annotation. We used pheatmap R package to determine gene copy numbers in the pathway with the parameter “scale = column”.

### Resequencing and SNP Calling

To reveal the adaptive mechanisms of *D. fruticosa* to the highest plateau, a total of 599 individuals from 25 populations were collected from north and southwest China (Fig. 2a; supplementary table S17, Supplementary Material online), including 21 highland populations across the QTP and 4 populations in northern China as a lowland control. Genomic DNAs were extracted from the fresh leaves by using the CTAB method (Porebski et al. 1997). The libraries were constructed with an insert size ranging from 300 to 500 bp and sequenced on the DNBSEQ-T1 platform of BGI-Shenzhen. Raw data were filtered by trimmomatic software with the parameters “ILLUMINACLIP: BGI_adapter.fa:2:35:4:12:true LEADING:3 TRAILING:3 SLIDINGWINDOW:5:15 MINLEN:50”.

After filtering low-quality bases, reads were mapped to the final assembly of *D. fruticosa* genome using Burrows-Wheeler Aligner (BWA) software (v.2.21) with the default parameters. The bam files were sorted and seven samples with an average depth of less than 10× were removed, and 592 individuals were used for further analyses (supplementary file S1, Supplementary Material online). PCR duplicates were marked by MarkDuplicates, and HaplotypeCaller was run on each bam file in a genomic variant call format (GVCF) mode. The 592 samples with an average depth of more than 10× were further consolidated into a single GVCF file, from which 84,530,065 SNPs and 31,407,195 small indels were identified using a joint calling approach. The SNPs and indels were further filtered using the following steps: (1) SNPs were filtered with “QD < 2.0| | FS > 60.0| | MQ < 40.0| | SOR > 3.0| | MQRankSum < −12.5| ReadPosRankSum < −8.0| | QUAL < 30.0”, and indels with “QD < 2.0| | FS > 200.0| | SOR > 10.0 | | MQRankSum < −12.5| ReadPosRankSum < −8.0”; resulting in 30,125,773 SNPs and 6,589,743 indels; (2) the variants were further filtered by vcftools (v. 0.1.13) with parameters: –max-missing 0.8 –maf 0.05 –minDP 3, resulting in 2,956,203 SNPs used for population genetic analyses; (3) SNPs with LD were pruned (−indep-pairwise 10 1 0.5) using PLINK (v. 1.9), resulting in 1,857,214 SNPs for clustering analysis.

All the variants were annotated using Annovar (Wang et al. 2010). SNPs and indels were categorized based on their positions on the chromosome (including intergenic regions, exons, introns, upstream regions, and downstream regions) and their effects (including nonsynonymous, synonymous, stopgain, stoploss, frameshift, nonframeshift) (supplementary table S22, Supplementary Material online).

### Population Genetic and Demographic History Analyses

PCA was performed on the filtered SNP set using GCTA (version 1.91.4beta3) (Yang et al. 2011). A neighbor-joining tree was constructed with 100 bootstraps using PHYLIP (version 3.696) (Felsenstein 2004) and the tree layout was generated using the online tool iTOL (http://itol.embl.de). The population structure was analyzed with the cluster number K ranging from 2 to 9 by ADMIXTURE (version1.3.0) (Alexander et al. 2009).

Genetic differentiation (*F*_ST_) was calculated with the parameters “–fst-window-size 50000 –fst-window-step 10000”, and nucleotide diversity (π) was inferred with the parameters “–window-pi 50000 –window-pi-step 10000” by using VCFtools (Danecek et al. 2011).

We identified ROH in all individuals using PLINK software (v. 1.9), which uses a sliding window approach to detect autozygous regions, without pruning the LD regions (Meyermans et al. 2020). Total 2,956,203 SNPs and 592 accessions were used for ROH identification with the parameters “–homozyg-density 50 –homozyg-gap 1000 –homozyg-kb 30 –homozyg-snp 40 –homozyg-window-het 2 –homozyg-window-snp 40 –homozyg-window-threshold 0.05 –homozyg-window-missing 10”. Historic population size was analyzed using the pairwise sequentially Markovian coalescent (PSMC) (Li and Durbin 2011) in 75 individuals from 25 populations (supplementary file S1, Supplementary Material online), with a sequence depth of more than 30×. First, we used bcftools mpileup with parameters “-C50 -Ou” to generate data sets, followed by bcftools call with parameters “-c -Ov” to call variants. Second, vcfutils.pl with parameters “vcf2fq -d 10 -D 100” was used to get consensus sequences. Third, fq2psmcfa with -q 20” was used to transform the consensus sequence into a fasta-like format. At last, PSMC with the parameters “-N25 -t10 -r5 -p “4 + 25*2 + 4 + 6' was used in each individual. In order to perform bootstrap analyses of different individuals from each clade, we first split the consensus sequence by using splitfa with the parameter “splitfa 100000”; then, the split consensus sequences were used for bootstrap analysis (100 replicates) by using PSMC software with parameters “-N25 -t10 -r5 -b -p 4 + 25*2 + 4 + 6”. Last, we plot the population size by combining the main PSMC result and 100 bootstrap results for each individual. An estimated mutation rate of 7.0 × 10^−9^ (Ossowski et al. 2010; Weng et al. 2019) and an average generation time of 3 years (based on field observations) were used to scale the population parameters into years and individuals.

### Selective Sweep Detection

To identify selective regions, we first scanned the high-altitude population group (clades II, III, IV, V) against the low-altitude population group (clade I). In particular, selection scans by comparing the central subclades (IVb, Vb) and plateau-edge subclades (IVa, Va) in two main clades (IV, V) were conducted, respectively. Then we calculated *F*_ST_ values between high-altitude population group and low-altitude population group with the parameters “–fst-window-size 50000 –fst-window-step 10000”, and between the two subclades (IVa vs. IVb, Va vs. Vb) respectively, with a top 5% cutoff used for further analysis. Second, XP-CLR test was performed on the high-altitude population group and low-altitude population group with the parameters “–ld 0.7 –maxsnps 400 –size 50000 –step 10000”, and the same analysis did among the two subclades, respectively. Third, we extracted the genes in the intersection of *F*_ST_ (top 5%) and XP-CLR (top 5%) between high-altitude population group and low-altitude population group, and the two subclades (IVa vs. IVb, Va vs. Vb), respectively. These overlap genes were further analyzed with function enrichment of GO terms by using EnrichPipeline.

In addition, we also performed selective sweep analysis using only the high-altitude population group using SweeD software (version 4.0.0) (Pavlidis et al. 2013) with the parameters “-minsnps 200 -maf 0.05” under a 50 kb slide window.

### Deleterious Mutations Identification

SNPs were annotated with SnpEff (v4.3t) (Cingolani et al. 2012). SIFT 4G (Vaser et al. 2016) was used to predict deleterious missense SNPs. Uniref90 (Suzek et al. 2007) was used as the reference database as recommended by the software. Missense SNPs with a score < 0.05 were defined as deleterious SNPs (dnSNPs), while SNPs with a score ≥ 0.05 were defined as tolerated SNPs (tnSNPs).

### Genetic Load Proxy Estimation

To estimate genetic load of different samples, we calculated the derived allele counts of dnSNPs predicted by SIFT 4G and 4-fold degenerate sites, respectively. The ratio of derived allele counts of dnSNPs and 4-fold degenerate sites was calculated for each sample and used as the proxy of genetic load, and referred to as estimated genetic load in this study.

### Ancestral State Inference

Ancestral state of each SNP site was determined based on a whole-genome alignment of *Dasiphora fruticosa* to *F. vesca* and *Rosa chinensis* using AnchorWave (Song et al. 2022). Alleles matched to the two outgroups were regarded as ancestral state, and those not matched but identical in two outgroups were defined as derived alleles.

## Supplementary Material

msae099_Supplementary_Data

## Data Availability

The sequencing raw data and genome assembly have been deposited into the CNGB Sequence Archive (CNSA) of China National GeneBank DataBase (CNGBdb) with accession number CNP0002342. The annotation files were uploaded on the figshare (https://figshare.com/s/46d2f2a30512240f3b37). Codes are available on Github (https://github.com/liumin4/analysis-of-Dasiphora-fruticosa/).
