# Supplementary material for: Signatures of Adaptation and Purifying Selection in Highland Populations of Dasiphora fruticosa
Source: Mol Biol Evol. 2024 May 20;41(6):msae099. doi: 10.1093/molbev/msae099 (PMC11156201; doi:10.1093/molbev/msae099)
Supplement: msae099_Supplementary_Data [file msae099_supplementary_data.zip › Supplementary Figures S1-S7 and Tables S1-S23-20240507.pdf]

Supplementary information for

Signatures of adaptation and purifying selection in highland populations of *Dasiphora fruticosa*

Fu-Sheng Yang,[1,3,4#] Min Liu,[2#] Xing Guo,[2#] Chao Xu,[1,3] Juan Jiang,[1,3,4] Weixue Mu,[2] Dongming Fang,[2] Yong-Chao Xu, [1,3] Fu-Min Zhang,[1,3,4] Ying-Hui Wang,[1,3,4] Ting Yang,[2] Hongyun Chen,[2] Sunil Kumar Sahu,[2] Ruirui Li,[2.4] Guanlong Wang,[2] Qiang Wang,[1,3,4] Xun Xu,[2] Song Ge,[1,3,4] Huan Liu,[2.4]* Ya-Long Guo[1,3,4]*

[1] State Key Laboratory of Systematic and Evolutionary Botany, Institute of Botany, Chinese Academy of Sciences, Beijing 100093, China

[2] State Key Laboratory of Agricultural Genomics, Key Laboratory of Genomics, Ministry of Agriculture, BGI Research, Shenzhen 518083, China

[3] China National Botanical Garden, Beijing 100093, China

[4] University of Chinese Academy of Sciences, Beijing 100049, China

[#] Contributed equally

* Correspondence: yalong.guo@ibcas.ac.cn or liuhuan@genomics.cn

Supplementary Information

Additional file 1

Supplementary Figs. S1-S7

Figure S1. *K*-mer frequency distribution of the *D. fruticosa* genome.

Figure S2. Genome-wide analysis of chromatin interactions in the *D. fruticosa* genome based on Hi-C data.

Figure S3. A chronogram showing the divergence time in Rosaceae and deep clades.

Figure S4. The pathways of carotenoid and ABA biosynthesis. **A**) The biosynthesis pathways of carotenoid and ABA. **B**) Gene copy number of the pathways in representative plants. The highlighted red circles represent gene expansion occurred in the pathway.

Figure S5. Proportions of gene families undergoing expansion and contraction in *D. fruticosa* and the nine reference plant species.

Figure S6. The intersection of F_{ST} and XP-CLR and candidate gene analyses. **A**) The intersection of F_{ST} and XP-CLR between the two subclade pairs (IVa vs IVb, Va vs Vb) in clades IV and V. **B**) The synteny analysis of *PME* genes between *D. fruticosa and Fragaria vesca*. **C**) A phylogenetic analysis of the *PME* gene family. **D**) The chromosome location of *PME* genes.

Figure S7. Summary results of ROH in each individual. **A**) Boxplot of total ROH (Mb) length in each individual. **B**) Distribution of the runs of homozygosity (ROH) length classes in each individual.

Supplementary Tables S1-S23

Table S1. Sequencing statistics for the *D. fruticosa* genome.

Table S2. Statistics of the nanopore assembly for the *D. fruticosa* genome.

Table S3. Statistics of the Hi-C assembly for the *D. fruticosa* genome.

Table S4. Statistics of chromosome level assembly for the *D. fruticosa* genome.

Table S5. Benchmarking Universal Single-Copy Orthologs (BUSCO) assessment.

Table S6. Mapping rates of the DNA and RNA reads.

Table S7. The annotation statistics of protein-coding genes in *D. fruticosa*, *Fragaria vesca*, and *Potentilla micrantha*.

Table S8. Summary of BUSCO assessment of protein-coding genes in *D. fruticosa*.

Table S9. Functional annotation of predicted genes in *D. fruticosa*.

Table S10. Summary of the transcription factor (TF) genes in *D. fruticosa* and the nine reference plant species.

Table S11. Summary of the annotation of ncRNA in *D. fruticosa* and *F. vesca*.

Table S12. Summary of the annotated TEs in *D. fruticosa* and *F. vesca*.

Table S13. Summary of gene family analysis of *D. fruticosa* and the nine reference plant species.

Table S14. Summary of the GO enrichment analysis of unique gene families in *D. fruticosa.*

Table S15. Gene copy number of MEP, carotenes, xanthophylls, ABA biosynthesis pathways in *D. fruticosa* and the nine reference species.

Table S16. The GO enrichment of the expanded and contracted genes.

Table S17. Population sampling.

Table S18. Summary of variant statistics.

Table S19. The average value of nucleotide diversity (π) of population groups.

Table S20. Summary of the GO enrichment analysis of candidate genes.

Table S21. F_{ST} values between clades of *D. fruticosa*.

Table S22. The annotation results of SNPs and indels.

Table S23. The total number of ROH in the individuals of five clades.

Additional file 2

Supplementary File S1-S10

Supplementary File S1. Sequencing statistics.

Supplementary File S2. The GO enrichment of the genes under selection in the high-altitude population group (clades II-V).

Supplementary File S3. The genes enriched in the three GOs in the high-altitude population group (clades II-V).

Supplementary File S4. The GO enrichment of the genes under selection in clade IVb against clade IVa population groups.

Supplementary File S5. The genes enriched in the four GOs in clade IVb against clade IVa population groups.

Supplementary File S6. The GO enrichment of the genes under selection in clade Vb against clade Va population groups.

Supplementary File S7. The genes which enriched in the GOs in clade Vb against clade Va population groups.

Supplementary File S8. The GO enrichment of the genes under selection shared in clade IVb and Vb populations.

Supplementary File S9. Frequency of stopgain SNP site of *POTFRU10221* gene in population groups.

Supplementary File S10. The SweeD result in the high-altitude population group.

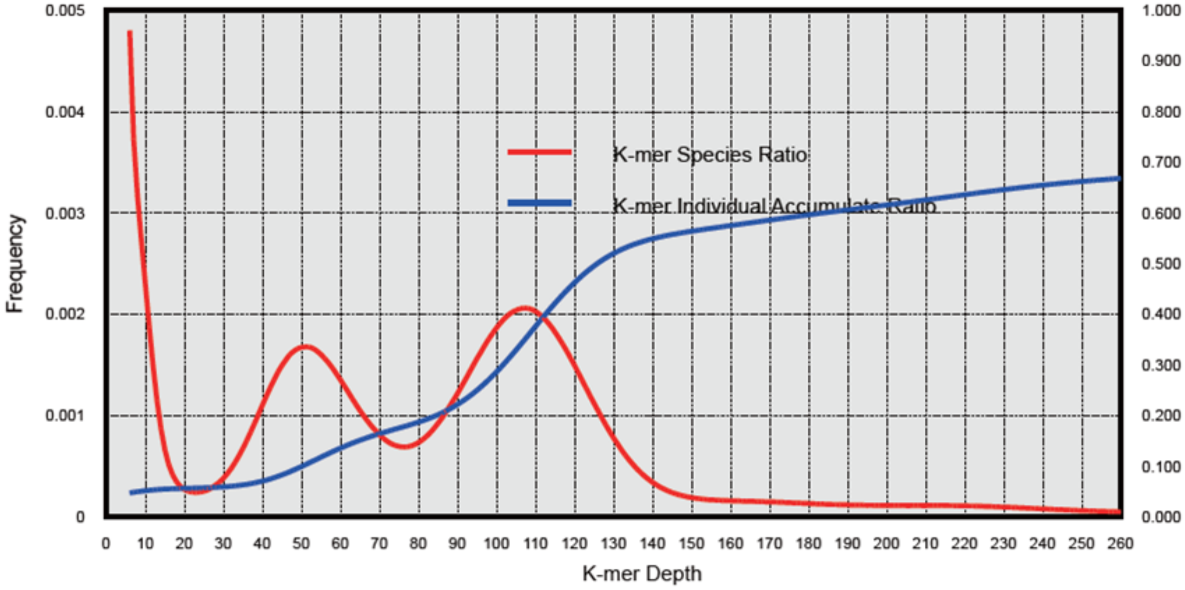

kmer	kmer num	pkdepth	genome size (Mb)	heterozygous ratio (%)	repeat ratio (%)
17	26,061,548,932	107	243,565,877	1.2	46.13

Figure S1. *K*-mer frequency distribution of the *D. fruticosa* genome.

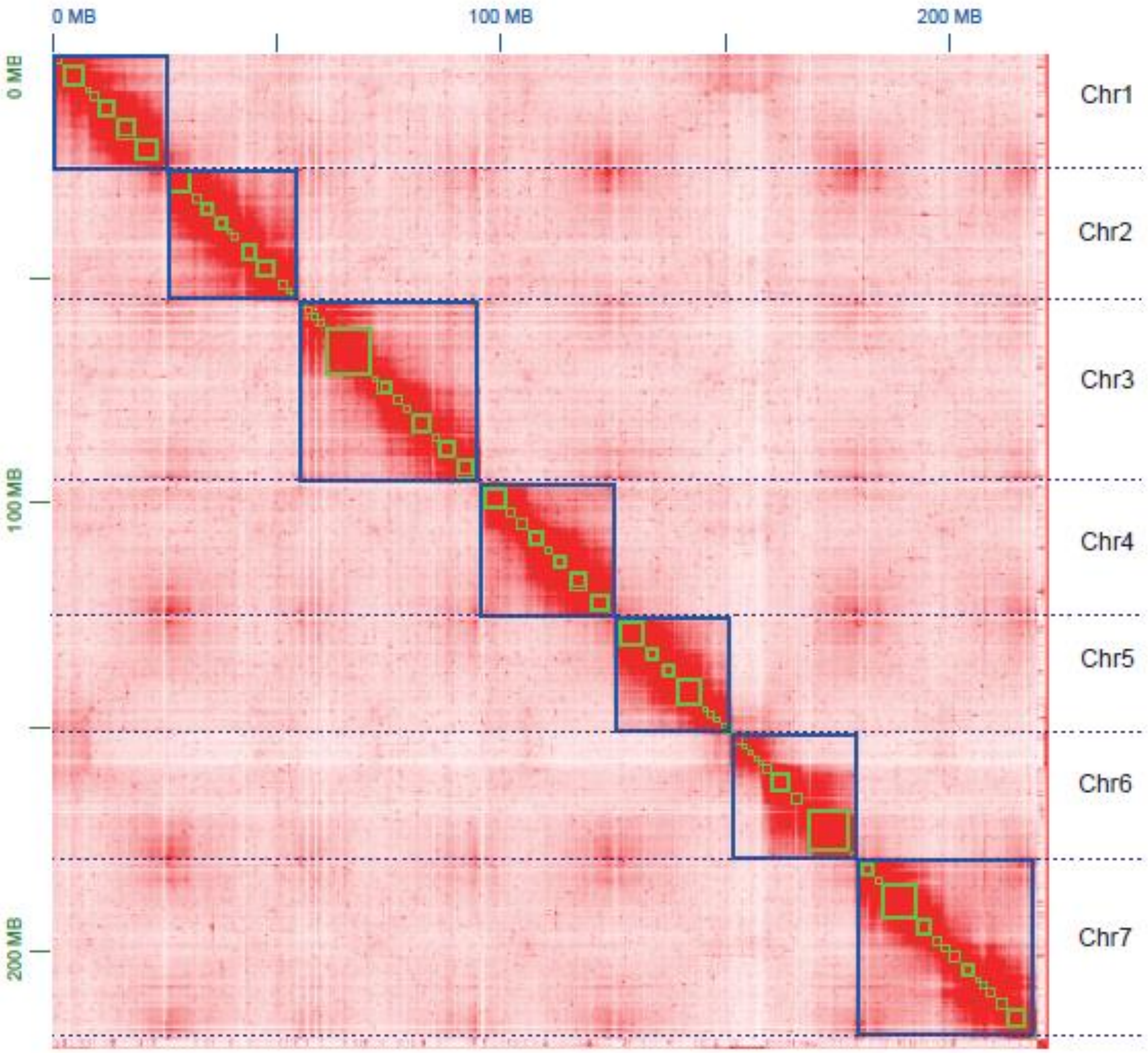

Figure S2. Genome-wide analysis of chromatin interactions in the *D. fruticosa* genome based on Hi-C data.

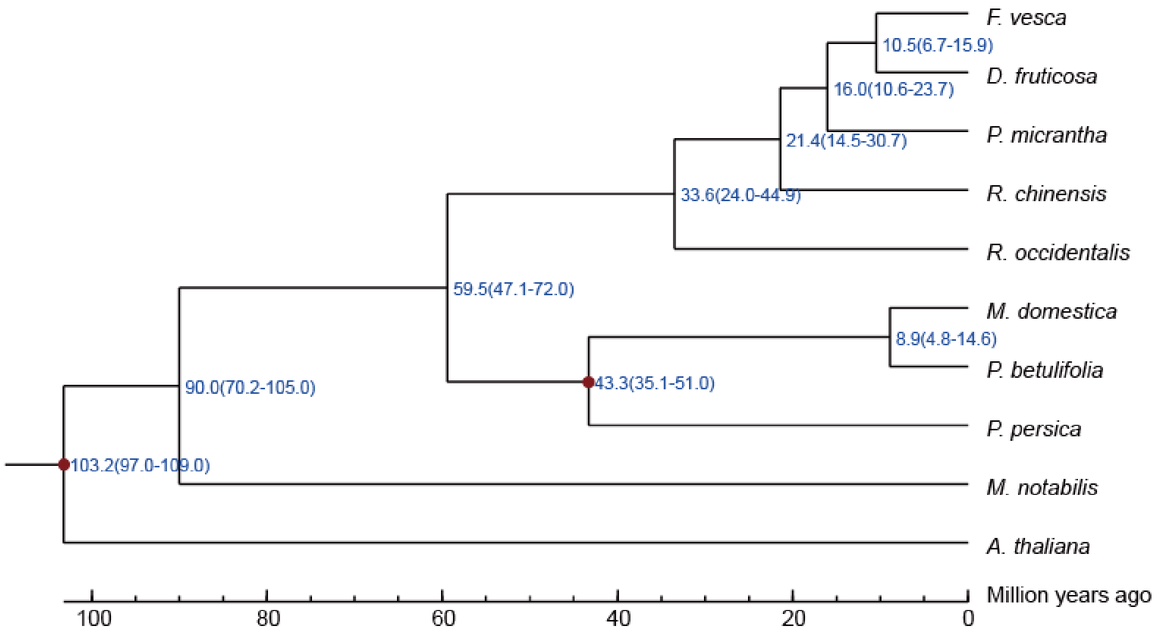

Figure S3. A chronogram showing the divergence time in Rosaceae and deep clades.

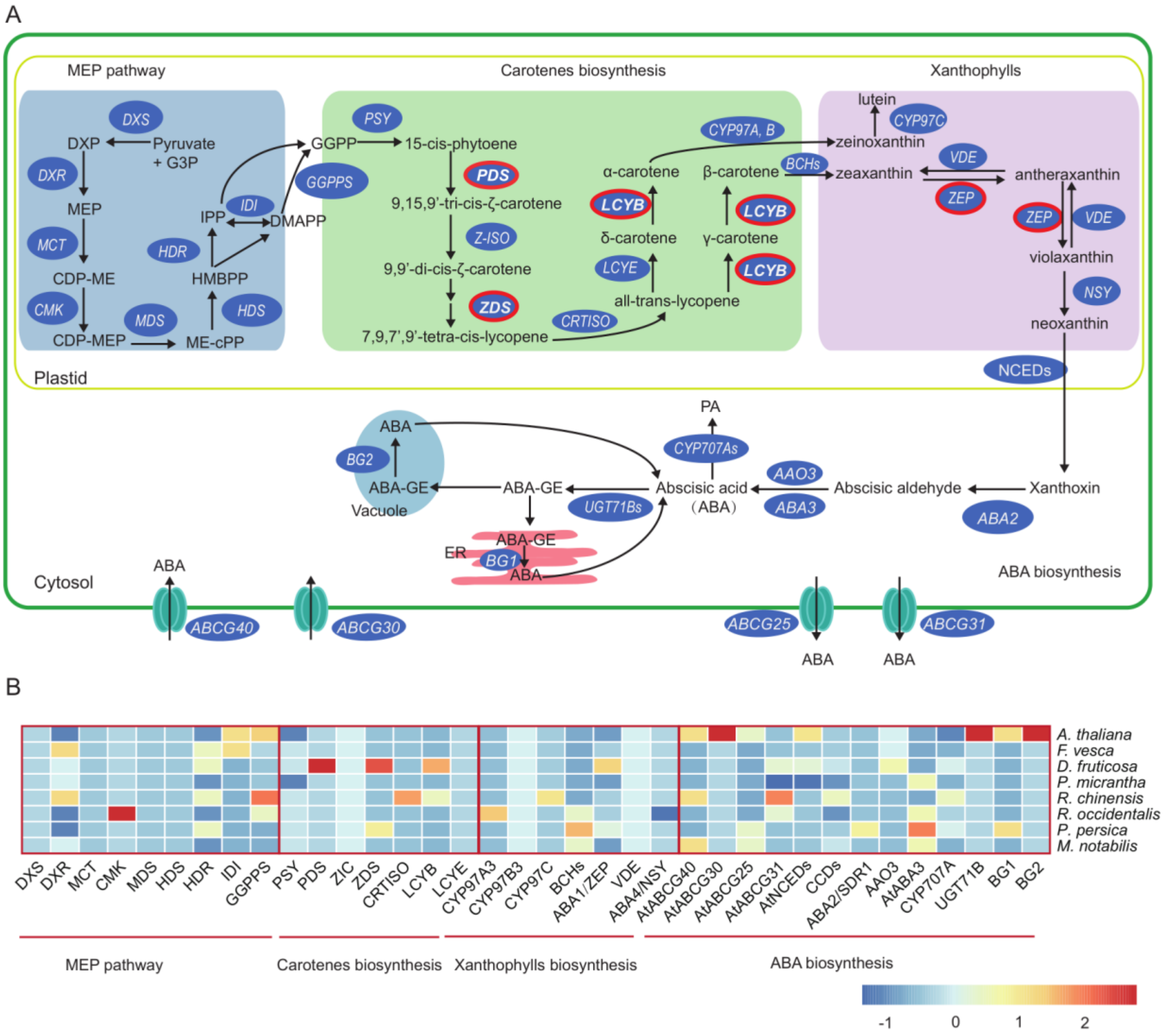

Figure S4. The pathways of carotenoid and ABA biosynthesis. **A)** The biosynthesis pathways of carotenoid and ABA. **B)** Gene copy number of the pathways in representative plants. The highlighted red circles represent gene expansion occurred in the pathway.

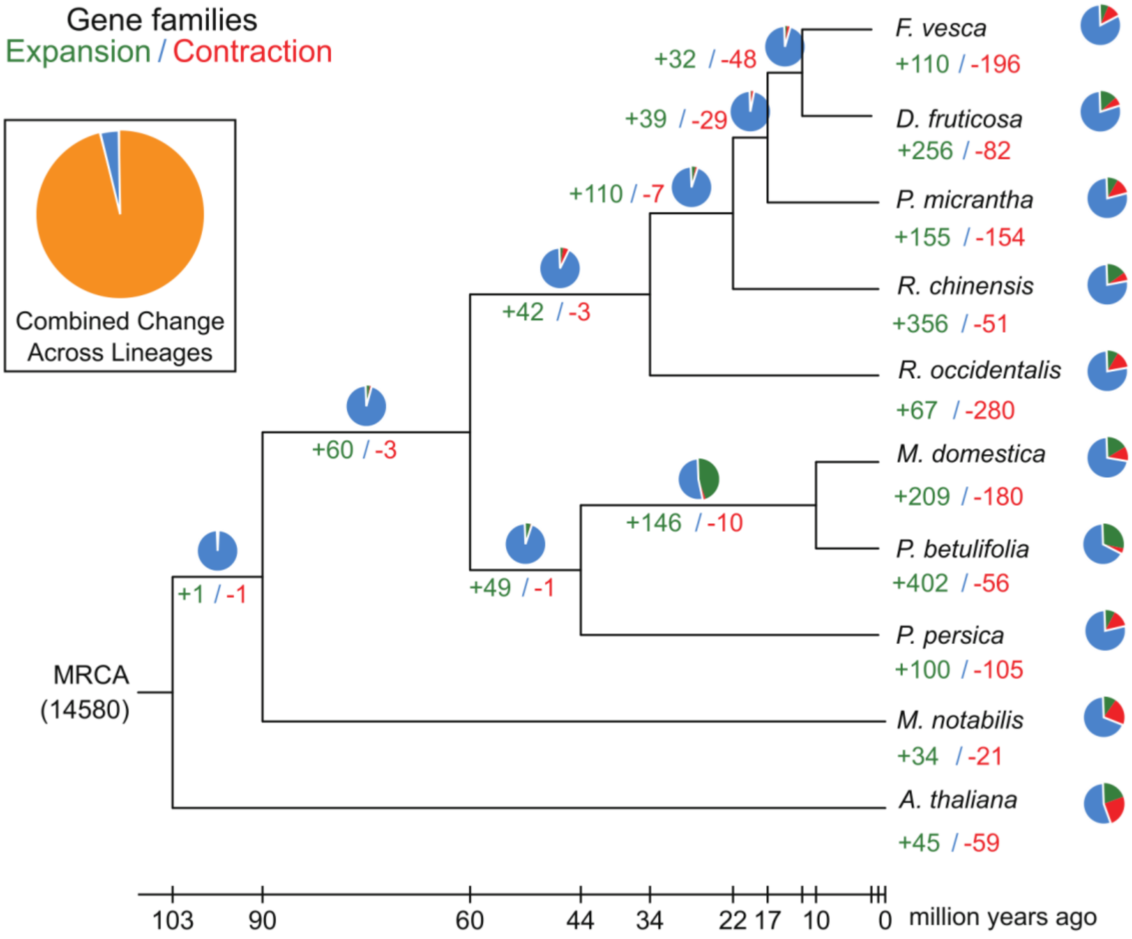

Figure S5. Proportions of gene families undergoing expansion and contraction in *D. fruticosa* and the nine reference plant species.

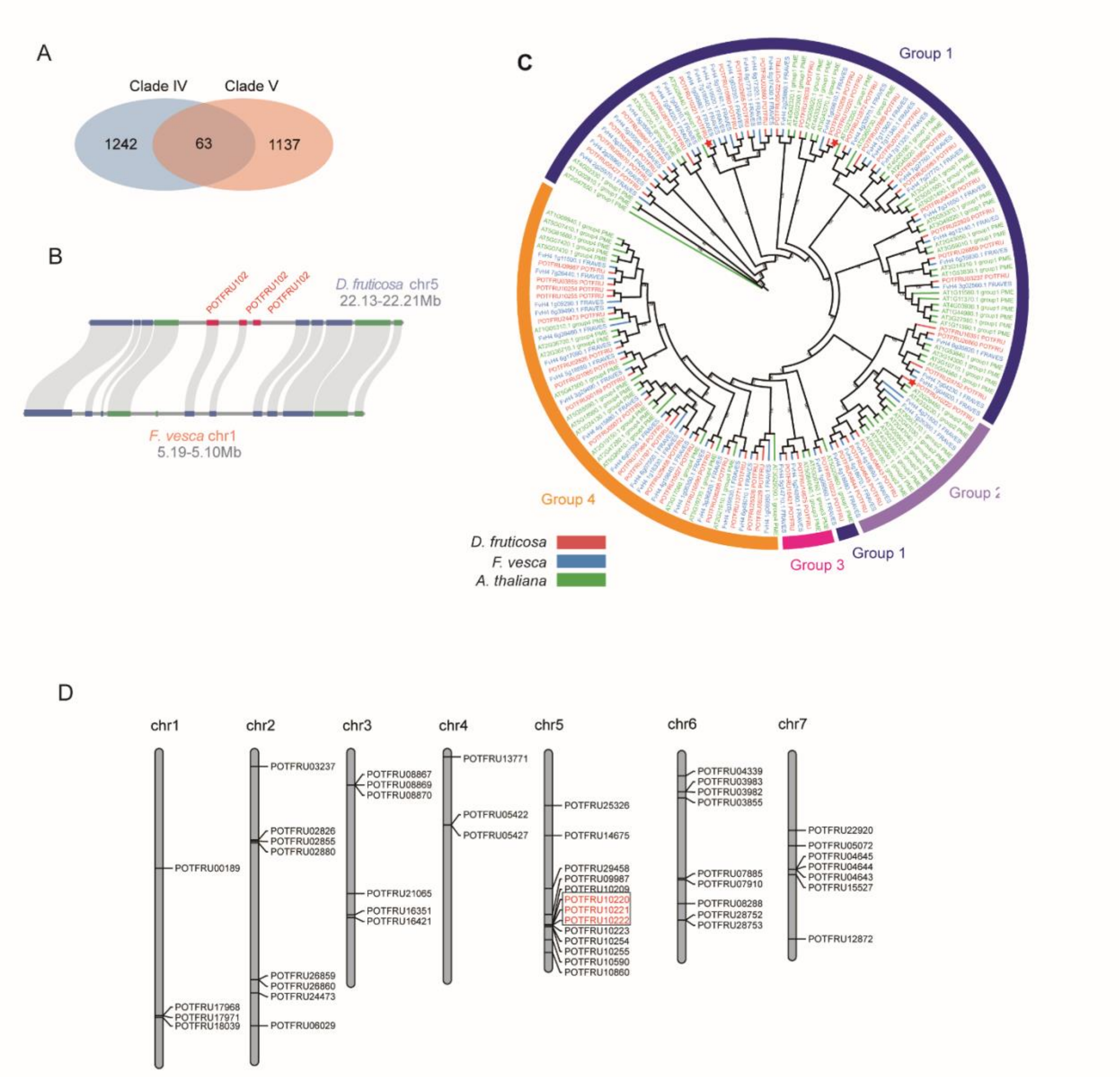

Figure S6. The intersection of F_{ST} and XP-CLR and candidate gene analyses. **A)** The intersection of F_{ST} and XP-CLR between the two subclade pairs (IVa vs IVb, Va vs Vb) in clades IV and V. **B)** The synteny analysis of *PME* genes between *D. fruticosa and Fragaria vesca*. **C)** A phylogenetic analysis of the *PME* gene family. **D)** The chromosome location of *PME* genes.

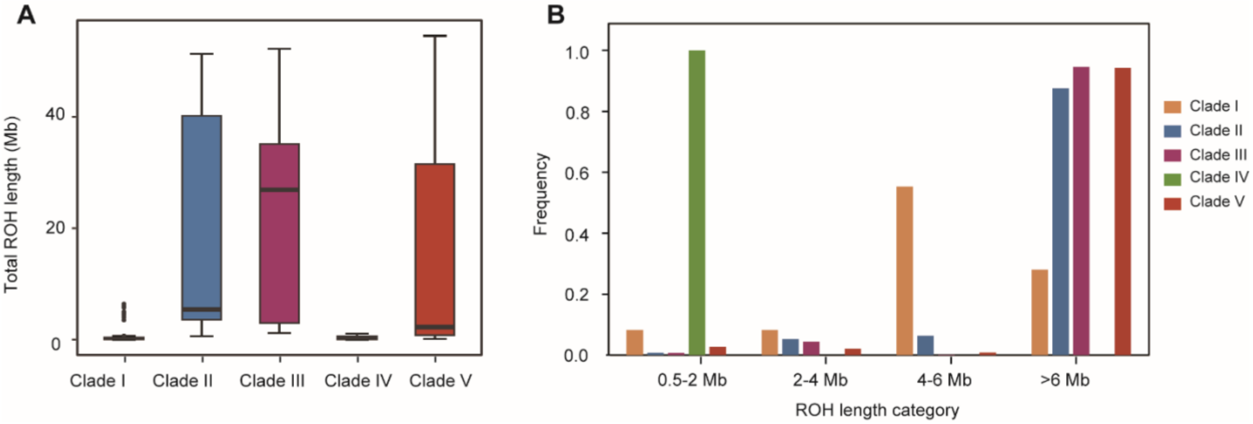

Figure S7. Summary results of ROH in each individual. **A)** Boxplot of total ROH (Mb) length in each individual. **B)** Distribution of the runs of homozygosity (ROH) length classes in each individual.

Table S1. Sequencing statistics for the *Dasiphora fruticosa* genome.

	Raw data			Clean data		
	Total number of reads (bp)	Total number of bases (bp)	Depth (×)	Total number of reads (bp)	Total number of bases (Gb)	Depth (×)
**WGS**	679,836,122	67,983,612,200	308	650,812,650	65,081,265,000	295
**Nanopore**	2,850,409	54,921,252,733	245	2,178,996	47,208,224,428	214
**HI-C**	501,833,998	100,366,799,600	454	NA	NA	NA
**RNA**	209,347,376	20,934,737,600	NA	204,451,040	20,444,864,740	NA

Table S2. Statistics of the nanopore assembly for the *D. fruticosa* genome.

Nanopore assembly, Maximal length (bp)	Contig Length (bp)	Number
	7,784,032	
**N90**	539,992	114
**N80**	866,852	83
**N70**	1,395,918	62
**N60**	1,750,099	48
**N50**	2,106,498	36
**N40**	2,449,088	26
**N30**	2,855,142	18
**N20**	3,310,836	11
**N10**	4,153,694	5
**Total length (bp)**	222,571,321	
**number>=100bp**		246
**number>=2kb**		246
**Ratio of Ns**	0.00%	

Table S3. Statistics of the Hi-C assembly for the *D. fruticosa* genome.

**Hi-C assembly**	**Scaffold Length (bp)**	**Number**	**Contig Length (bp)**	**Number**
**Maximal length (bp)**	40,277,584		7,784,032	
**N90**	26,106,998	7	515,235	119
**N80**	26,435,175	6	828,952	85
**N70**	27,726,264	5	1,353,049	62
**N60**	29,396,715	4	1,750,099	48
**N50**	**29,396,715**	**4**	**2,073,588**	**37**
**N40**	29,815,186	3	2,368,491	27
**N30**	39,920,505	2	2,855,142	18
**N20**	39,920,505	2	3,275,796	11
**N10**	40,277,584	1	4,153,694	5
**Total length (bp)**	222,633,563		222,571,260	
**number>=100bp**		89		332
**number>=2kb**		72		314
**Ratio of Ns**	0.03%			

Table S4. Statistics of chromosome level assembly for the *D. fruticosa* genome.

Chromosome	Length of chromosome (bp)	G+C contents (%)
**chr1**	40,277,584	38.99%
**chr2**	39,920,505	39.00%
**chr3**	29,815,186	38.90%
**chr4**	29,396,715	38.96%
**chr5**	27,726,264	39.15%
**chr6**	26,435,175	38.97%
**chr7**	26,106,998	38.94%
**Total**	219,678,427	38.99%

Table S5. Benchmarking Universal Single-Copy Orthologs (BUSCO) assessment.

	**Number**	**Percentage (%)**
**Complete BUSCOs**	1,341	97.5
**Complete and single-copy BUSCOs (S)**	1,282	93.2
**Complete and duplicated BUSCOs (D)**	59	4.3
**Fragmented BUSCOs (F)**	8	0.6
**Missing BUSCOs (M)**	26	1.9
**Toal BUSCO groups searched**	1,375	100

Table S6. Mapping rates of the DNA and RNA reads.

Species	Total DNA reads mapped rate (%)	Properly DNA paired reads mapped rate (%)	Overall RNA reads alignment rate (%)
D. fruticosa	97.28%	88.99%	90.88%

Table S7. The annotation statistics of protein-coding genes in *D. fruticosa*, *Fragaria vesca*, and *Potentilla micrantha*.

Species	Gene number	Mean mRNA length (bp)	Mean coding sequence length (bp)	Mean exons per gene	Mean exon length (bp)	Mean intron length (bp)
D. fruticosa	31,351	2887.92	1082.62	4.58	236.05	384.22
F. vesca	24,858	2733.45	1261.68	5.33	236.52	339.55
P. micrantha	33,602	3214.63	1148.58	5.14	223.27	498.51

Table S8. Summary of BUSCO assessment of protein-coding genes in *D. fruticosa*

Type	*D. fruticosa*
**Complete BUSCOs**	1,290 (93.9%)
**Complete and single-copy BUSCOs**	1,248 (90.8%)
**Complete and duplicated BUSCOs**	42 (3.1%)
**Fragmented BUSCOs**	30 (2.3%)
**Missing BUSCOs**	55 (3.9%)
**Total BUSCO groups searched**	1,375 (100%)

Table S9. Functional annotation of predicted genes in *D. fruticosa*.

Item	Annotated Num	Annotated Percent (%)
**NR**	27,854	88.85%
**Swiss-Prot**	22,144	70.63%
**KEGG**	21,708	69.24%
**KOG**	21,131	67.40%
**InterPro**	28,298	90.26%
**Annotated**	29,389	93.74%
**Total**	31,351	100%

Table S10. Summary of the transcription factor (TF) genes in *D. fruticosa* and the nine reference plant species.

	A. thaliana	*F. vesca*	*M. domestica*	*M. notabilis*	*D. fruticosa*	*P. micrantha*	*P. persica*	*P. betulifolia*	*R. chinensis*	*R. occidentalis*
**AP2-ERF-RAV**	182	142	258	136	**149**	92	153	281	162	146
**ARF**	48	35	64	31	**37**	33	30	80	33	36
**ARR-B**	74	57	106	61	**64**	58	63	110	62	67
**B3**	20	25	31	17	**23**	28	16	36	38	18
**BBR-BPC**	7	3	6	3	**4**	3	3	6	3	3
**BES1**	8	6	19	7	**7**	8	7	13	9	7
**bHLH**	119	88	163	86	**87**	82	96	171	91	89
**bZIP**	66	42	77	48	**50**	46	51	103	50	46
**C2H2**	8	4	3	3	**4**	5	2	5	5	5
**C3H**	0	1	2	1	**1**	0	0	1	0	0
**CAMTA**	2	4	11	4	**5**	6	2	7	5	2
**CO-like**	52	39	70	40	**38**	37	38	72	43	40
**CPP**	8	6	11	8	**6**	6	6	13	5	6
**DBB**	28	19	40	19	**20**	20	20	40	19	20
**Dof**	36	23	44	24	**23**	25	25	47	21	24
**E2F_or_DP**	8	7	10	6	**8**	7	7	11	11	8
**EIL**	6	4	11	6	**4**	5	5	11	3	4
**FAR1**	17	0	14	12	**56**	57	64	49	32	41
**G2-like**	55	39	77	44	**43**	43	42	81	41	41
**GATA**	28	19	32	23	**20**	19	20	39	18	15
**GeBP**	22	4	9	11	**6**	8	8	12	5	8
**GRAS**	34	46	65	42	**52**	43	46	98	56	40
**GRF**	9	10	16	10	**14**	11	9	16	10	11
**HB-other**	18	13	24	14	**15**	9	11	26	12	14
**HB-PHD**	16	9	18	13	**12**	11	10	20	11	10
**HD-ZIP**	48	31	67	33	**32**	31	30	68	30	37

**HRT-like**	3	1	1	1	**3**	2	1	2	3	0
**HSF**	24	18	37	21	**16**	18	19	36	21	19
**LBD**	43	34	59	33	**39**	21	37	69	38	40
**LFY**	1	4	2	1	**0**	1	2	2	1	1
**LSD**	3	3	5	3	**6**	2	2	4	3	3
**MIKC_MADS**	80	67	124	63	**73**	75	77	117	79	62
**M-type_MADS**	107	69	120	55	**70**	79	77	118	76	63
**MYB**	137	122	213	112	**115**	112	69	215	135	116
**MYB_related**	8	9	16	8	**8**	6	9	20	9	7
**NAC**	110	89	169	76	**112**	112	72	182	110	89
**NF-X1**	1	1	1	1	**1**	1	1	2	1	1
**NF-YA**	10	6	11	7	**6**	6	6	13	7	7
**NF-YB**	13	14	25	15	**13**	13	14	25	13	13
**NF-YC**	14	9	13	8	**9**	9	11	17	10	10
**Nin-like**	14	6	13	7	**10**	8	9	15	8	9
**NZZ_or_SPL**	3	2	6	3	**2**	2	3	5	3	1
**S1Fa-like**	4	2	3	3	**2**	2	2	5	2	2
**SAP**	1	1	2	1	**1**	1	1	2	1	1
**SBP**	17	16	32	15	**15**	18	17	34	16	15
**SRS**	11	6	11	5	**5**	6	6	11	6	5
**STAT**	0	0	0	0	**1**	0	0	0	2	1
**TALE**	21	19	38	21	**19**	19	20	38	21	19
**TCP**	24	17	34	22	**17**	19	21	38	17	17
**Trihelix**	27	29	52	27	**30**	34	30	53	28	30
**VOZ**	2	2	6	2	**2**	2	2	4	2	2
**Whirly**	3	2	4	1	**2**	2	2	4	2	3
**WOX**	16	16	18	11	**13**	12	9	18	207	11
**WRKY**	72	60	112	55	**64**	57	59	117	66	56
**YABBY**	6	6	12	6	**6**	6	6	12	6	6

**ZF-HD**	17	10	17	10	**10**	9	10	24	10	10
**Total**	1,711	1,316	2,404	1,295	**1,450**	1,347	1,358	2,618	1,678	1,357

Table S11. Summary of the annotation of ncRNA in *D. fruticosa* and *F. vesca*.

		D. fruticosa				*F. vesca*			
**Type**		**Copy (w)**	**Average length(bp)**	**Total length(bp)**	**% of genome**	**Copy (w)**	**Average length(bp)**	**Total length(bp)**	**% of genome**
**miRNA**		103	124.65	12,839	0.005768	83	125.3	10,400	0.00472
**tRNA**		543	74.91	40,676	0.018275	495	74.83	37,039	0.016809
**rRNA**	rRNA	690	161.59	111,502	0.050097	271	287.63	77,949	0.035374
	18S	45	827.71	37,247	0.016735	40	1,181.47	47,259	0.021447
	28S	90	130.5	11,745	0.005277	122	140.47	17,138	0.007777
	5.8S	23	139.13	3,200	0.001438	30	146.13	4,384	0.001989
	5S	532	111.48	59,310	0.026647	79	116.05	9,168	0.004161
**snRNA**	snRNA	405	118.76	48,100	0.021611	414	114.55	47,424	0.021521
	CD-box	195	95.44	18,612	0.008362	223	97.63	21,772	0.00988
	HACA-box	44	128.61	5,659	0.002543	47	122.55	5,760	0.002614
	splicing	166	142.55	23,829	0.010706	144	138.14	19,892	0.009027

Table S12. Summary of the annotated TEs in *D. fruticosa* and *F. vesca*.

**Transposable elements (TEs)**		***D. fruticosa***		***F. vesca***	
**Repeat elements**	**Type**	**% in genome**	**Length(bp)**	**% in genome**	**Length (bp)**
**Type I: Retrotransposon elements**	SINE	0.01	14,063	0.03	75,025
	LINE	5.47	12,168,022	1.93	4,249,005
	LTR	21.36	47,563,458	22.55	49,689,236
	Copia	9.32	20,741,434	8.77	19,321,767
	Gypsy	8.25	18,363,597	11.81	26,030,420
**Type II: DNA transposon**	DNA	14.69	32,696,425	13.33	29,365,674
**Type III: Tandem repeats**	Satellite	0.76	1,690,061	0.06	140,681
	Simple_repeat	0.45	1,009,101	0.02	47,100
**Unknown**		3.25	7,250,480	2.59	5,716,566
**Total repeat**		41.09	91,450,270	35.73	78,723,395

Table S13. Summary of gene family analysis of *D. fruticosa* and the nine reference plant species.

Species	Genes number	Genes in families	Unclustered genes	Family number	Unique families	Average genes per family
A. thaliana	27,416	24,738	2,678	13,557	882	1.82
M. notabilis	26,965	23,014	3,951	14,705	792	1.57
M. domestica	44,677	41,749	2,928	17,131	863	2
P. betulifolia	59,552	55,293	4,259	17,580	727	3.15
P. persica	26,335	24,696	1,639	15,282	467	1.62
R. chinensis	39,669	33,080	6,589	17,055	1,104	1.94
R. occidentalis	27,612	23,478	4,134	15,643	531	1.5
F. vesca	24,858	23,190	1,668	15,227	267	1.52
D. fruticosa	**31,351**	**29,138**	**2,214**	**16,699**	**677**	**1.74**
P. micrantha	32,763	29,086	3,677	16,166	766	1.8

Table S14. Summary of the GO enrichment analysis of unique gene families in *D. fruticosa*.

GO ID	GO Term	GO Class	P-value	Adjusted P-value	Gene number	GO level
**GO:0022402**	cell cycle process	BP	9.90E-10	2.63E-07	41	3
**GO:1901177**	**lycopene biosynthetic process**	BP	6.64E-09	9.30E-07	6	8
**GO:0061077**	chaperone-mediated protein folding	BP	8.11E-08	8.30E-06	13	4
**GO:0006302**	double-strand break repair	BP	9.28E-08	9.15E-06	18	7
**GO:0006310**	DNA recombination	BP	1.01E-07	9.55E-06	21	6
**GO:0000056**	ribosomal small subunit export from nucleus	BP	3.93E-07	3.08E-05	6	7
**GO:0000730**	DNA recombinase assembly	BP	4.46E-07	3.21E-05	8	7
**GO:0042148**	strand invasion	BP	8.97E-07	6.12E-05	8	6
**GO:0000055**	ribosomal large subunit export from nucleus	BP	1.38E-06	8.56E-05	6	7
**GO:0051026**	chiasma assembly	BP	4.80E-06	0.000284	7	4
**GO:0046246**	terpene biosynthetic process	BP	2.01E-05	0.0009381	8	6
**GO:0016117**	**carotenoid biosynthetic process**	BP	2.46E-05	0.0010905	8	6
**GO:0010212**	response to ionizing radiation	BP	2.99E-05	0.0012844	10	5
**GO:0010236**	plastoquinone biosynthetic process	BP	3.22E-05	0.0013173	4	7
**GO:0033234**	negative regulation of protein sumoylation	BP	3.22E-05	0.0013173	4	8
**GO:0046825**	regulation of protein export from nucleus	BP	3.53E-05	0.0014217	6	7
**GO:0006913**	nucleocytoplasmic transport	BP	4.03E-05	0.0015549	15	6
**GO:0046822**	regulation of nucleocytoplasmic transport	BP	5.15E-05	0.0019309	7	6
**GO:0070192**	chromosome organization involved in meiotic cell cycle	BP	5.84E-05	0.0021563	10	4
**GO:0051223**	regulation of protein transport	BP	6.62E-05	0.002229	10	6
**GO:0046686**	response to cadmium ion	BP	8.99E-05	0.0028141	24	6
**GO:0098813**	nuclear chromosome segregation	BP	0.0001198	0.0035418	15	4
**GO:0008104**	protein localization	BP	0.0001277	0.0037315	34	4
**GO:0006222**	UMP biosynthetic process	BP	0.0001405	0.0039347	5	9
**GO:0015031**	protein transport	BP	0.0001734	0.0046578	28	5

**GO:0033036**	macromolecule localization	BP	0.0001789	0.0047593	40	3
**GO:0043097**	pyrimidine nucleoside salvage	BP	0.0001895	0.004895	5	6
**GO:0010501**	RNA secondary structure unwinding	BP	0.00023	0.005827	10	6
**GO:0071705**	nitrogen compound transport	BP	0.0002385	0.0059859	37	5
**GO:0001708**	cell fate specification	BP	0.0002472	0.0061445	9	5
**GO:0006370**	7-methylguanosine mRNA capping	BP	0.0002743	0.0066329	4	8
**GO:0045132**	meiotic chromosome segregation	BP	0.0002816	0.0067476	10	4
**GO:0051049**	regulation of transport	BP	0.0005552	0.0116252	16	4
**GO:0033157**	regulation of intracellular protein transport	BP	0.0005594	0.0116252	7	6
**GO:0009112**	nucleobase metabolic process	BP	0.0005594	0.0116252	7	5
**GO:0006886**	intracellular protein transport	BP	0.000624	0.0128676	24	5
**GO:0009846**	pollen germination	BP	0.000706	0.0141196	9	4
**GO:0071702**	organic substance transport	BP	0.0008028	0.0150575	43	5
**GO:0045037**	protein import into chloroplast stroma	BP	0.0008038	0.0150575	5	7
**GO:0034613**	cellular protein localization	BP	0.0008174	0.0152041	28	5
**GO:0009119**	ribonucleoside metabolic process	BP	0.0008275	0.0152859	7	6
**GO:0065004**	protein-DNA complex assembly	BP	0.0008356	0.0153285	10	6
**GO:0007076**	mitotic chromosome condensation	BP	0.0010041	0.0179251	3	5
**GO:0071824**	protein-DNA complex subunit organization	BP	0.0012915	0.0217658	11	5
**GO:1900150**	regulation of defense response to fungus	BP	0.0013096	0.0217658	6	4
**GO:0000706**	meiotic DNA double-strand break processing	BP	0.0013338	0.0217658	4	4
**GO:0010038**	response to metal ion	BP	0.0015149	0.024422	28	5
**GO:0006419**	alanyl-tRNA aminoacylation	BP	0.0015689	0.0248402	3	8
**GO:0006405**	RNA export from nucleus	BP	0.0017009	0.0266145	9	6
**GO:0006974**	**cellular response to DNA damage stimulus**	BP	0.0017326	0.0267947	25	5
**GO:0017038**	protein import	BP	0.0019283	0.0288168	11	5
**GO:0000354**	cis assembly of pre-catalytic spliceosome	BP	0.0022982	0.0329658	3	6
**GO:0006206**	pyrimidine nucleobase metabolic process	BP	0.0026787	0.0342946	5	6
**GO:0060341**	regulation of cellular localization	BP	0.0027018	0.0342946	9	4

**GO:0051649**	establishment of localization in cell	BP	0.002776	0.0342946	31	4
**GO:0048484**	enteric nervous system development	BP	0.0029395	0.0342946	2	5
**GO:0042882**	L-arabinose transport	BP	0.0029395	0.0342946	2	9
**GO:0031503**	protein complex localization	BP	0.0032015	0.0358365	8	5
**GO:0010043**	response to zinc ion	BP	0.0034847	0.0379885	6	6
**GO:0009553**	embryo sac development	BP	0.0035751	0.0386571	13	6
**GO:0006281**	**DNA repair**	BP	0.0035909	0.0386713	21	6
**GO:0009697**	salicylic acid biosynthetic process	BP	0.0038698	0.0408881	4	7
**GO:0010071**	root meristem specification	BP	0.004306	0.044395	3	4
**GO:0080009**	mRNA methylation	BP	0.004306	0.044395	3	6
**GO:0071426**	ribonucleoprotein complex export from nucleus	BP	0.0043632	0.0446389	8	5

Table S15. Gene copy number of MEP, carotenes, xanthophylls, ABA biosynthesis pathways in *D. fruticosa* and the nine reference species.

Item	Gene name	*A. thaliana*	*F. vesca*	*D. fruticosa*	*P. micrantha*	*R. chinensis*	*R. occidentalis*	*M. domestica*	*P. persica*	*P. betulifolia*	*M. notabilis*
**MEP pathway**	**DXS**	1	1	1	1	1	1	1	1	2	1
	**DXR**	1	3	2	2	3	1	2	1	3	2
	**MCT**	1	1	1	1	1	1	2	1	2	1
	**CMK**	1	1	1	1	1	2	1	1	1	1
	**MDS**	1	1	1	1	1	1	2	1	2	1
	**HDS**	1	1	1	1	1	1	2	1	2	1
	**HDR**	1	2	2	1	2	1	3	2	2	1
	**IDI**	2	2	1	1	1	1	2	1	2	1
	**GGPPS**	11	4	3	3	14	7	2	2	5	2
**Carotenes biosynthesis**	**PSY**	1	3	3	1	3	3	6	3	7	3
	**PDS**	1	1	**3**	1	1	1	1	1	1	1
	**Z-ISO**	1	1	1	1	1	1	1	1	1	1
	**ZDS**	1	1	**7**	1	1	1	2	4	3	1
	**CRTISO**	2	2	2	2	4	2	4	2	3	2
	**LCYB**	1	1	**2**	1	2	1	3	1	2	1
	**LCYE**	1	1	1	1	1	1	1	1	3	1
**Xanthophylls biosynthesis**	**CYP97A3**	1	1	1	1	1	2	2	1	2	1
	**CYP97B3**	1	1	1	1	1	1	1	1	1	1
	**CYP97C**	1	1	1	1	2	1	1	1	3	1
	**BCHs**	2	2	2	2	2	3	4	4	3	3
	**ABA1/NPQ2/ZEP**	1	1	**4**	1	2	2	4	3	4	1
	**NPQ1/VDE**	1	1	**1**	1	1	1	1	1	1	1
	**ABA4/NSY**	1	1	1	1	1	0	3	1	3	1
**ABA biosynthes**	**AtABCG40**	1	0	0	0	1	0	1	0	0	1
	**AtABCG30**	1	0	0	0	0	0	0	0	0	0
	**AtABCG25**	1	0	0	0	0	0	2	1	2	1

**AtABCG31**	1	1	2	0	4	2	2	1	3	1
**AtNCEDs**	5	3	4	2	3	3	6	3	5	3
**CCDs**	4	4	5	2	7	2	9	4	13	5
**ABA2/SDR1**	1	1	1	1	1	1	2	2	3	1
**AAO3**	1	1	2	0	0	0	1	0	5	0
**AtABA3**	1	1	1	2	1	2	2	3	1	2
**CYP707A**	5	6	8	6	9	7	12	8	12	6
**UGT71B**	1	0	0	0	0	0	0	0	0	0
**BG1**	1	0	0	0	0	0	1	1	1	0
**BG2**	1	0	0	0	0	0	0	0	0	0

Table S16. The GO enrichment of the expanded and contracted genes.

GO ID	GO Term	GO Class	P-value	Adjusted P-value	Gene number	GO level
**GO:0007165**	signal transduction	BP	8.39E-81	4.03E-79	81	3
**GO:0051716**	cellular response to stimulus	BP	1.63E-24	3.12E-23	84	3
**GO:0009628**	**response to abiotic stimulus**	BP	0.001437	0.004928	6	3
**GO:0050794**	regulation of cellular process	BP	0.006575	0.017535	115	3
**GO:0050789**	regulation of biological process	BP	0.014803	0.030893	115	3
**GO:0034457**	Mpp10 complex	CC	0.000618	0.002199	3	3
**GO:0005838**	proteasome regulatory particle	CC	0.007265	0.017882	2	3
**GO:0008541**	proteasome regulatory particle, lid subcomplex	CC	0.007265	0.017882	2	3
**GO:0022624**	proteasome accessory complex	CC	0.007265	0.017882	2	3
**GO:0099081**	supramolecular polymer	CC	0.011597	0.026507	5	3
**GO:0044421**	extracellular region part	CC	0.020121	0.040241	4	3
**GO:0031012**	extracellular matrix	CC	0.020121	0.040241	4	3
**GO:0005515**	protein binding	MF	5.83E-118	5.59E-116	506	3
**GO:0140098**	catalytic activity, acting on RNA	MF	6.13E-09	5.35E-08	49	3
**GO:0097367**	carbohydrate derivative binding	MF	0.000304	0.001168	240	3
**GO:0005199**	**structural constituent of cell wall**	**MF**	**0.002316**	**0.007667**	**3**	**3**
**GO:0036094**	small molecule binding	MF	0.003336	0.010145	254	3
**GO:0005200**	structural constituent of cytoskeleton	MF	0.006494	0.017535	5	3
**GO:0043167**	ion binding	MF	0.008312	0.019463	368	3

Table S 17. Population sampling.

Populations	Sample name	Number	Latitude	Longitude	Altitude (m)
**QH1**	PF_2	26	36.70	101.26	2680
**QH2**	PF_3	22	36.90	100.94	2940
**SCKD**	PF_4	27	30.14	101.79	4410
**YNDQ**	PF_5	15	28.39	99.00	4512
**YNSR**	PF_6	21	28.15	99.90	4478
**SX1**	PF_7	30	39.04	113.55	2300
**SX2**	PF_8	29	39.08	113.57	2450
**XZLS**	PF_9	33	29.77	91.18	4461
**XZSGT**	PF_13	25	28.99	88.78	4549
**XZGB**	PF_14	25	28.40	88.76	4543
**XZYD1**	PF_15	24	27.85	88.87	4500
**XZYD2**	PF_16	22	28.58	87.43	4197
**XZZF**	PF_17	30	28.14	86.85	5121
**XZTR**	PF_18	24	28.10	87.35	4100
**XZNY1**	PF_19	25	28.59	86.51	4510
**XZNY2**	PF_20	26	28.14	85.96	4150
**XZNY3**	PF_21	24	28.52	86.17	5098
**XZZB**	PF_22	25	29.64	84.24	4720
**XZSG**	PF_23	24	28.81	85.51	4560
**XZPR**	PF_24	25	31.01	81.32	4950
**SAX**	PF_25	23	33.99	107.80	3000
**SCLD**	PF_30	14	29.55	101.97	3500
**SCYJ**	PF_31	25	30.06	101.30	3600
**XZNC**	PF_WY	26	29.61	94.63	4600
**INM**	PF_HW	9	43.62	118.56	1500
**Total**		599			

Table S18. Summary of variant statistics.

chromosome	raw SNPs	raw indels	hard filtered SNPs	hard filtered indels	after filter missing rate and MAF	after filter LD
**chr1**	16049836	6013872	5430625	1188317	477998	312403
**chr2**	15444226	5792904	5543491	1219724	576149	358262
**chr3**	11609385	4354889	4127192	905902	402276	250342
**chr4**	11095423	4157411	4093128	906831	434206	262517
**chr5**	10115170	3525066	3687681	771598	343862	222458
**chr6**	10450968	3917263	3614596	790480	322405	211420
**chr7**	9765057	3645790	3629060	806891	399307	239812
**total**	84530065	31407195	30125773	6589743	2956203	1857214

Table S19. The average value of nucleotide diversity (π) of population groups.

	π value				Tajima D			
	**mean**	**median**	**std**	**p-value**	**mean**	**median**	**std**	**p-value**
**Low altitude (clade I)**	0.002913	0.002931	0.001077	4.13974E-30	1.790219	1.780340	0.565482	0.000000
**High altitude (clade II-V)**	0.003031	0.003095	0.001094		2.276847	2.256175	0.406813	
**clade IVa**	0.002878	0.002872	0.001193	0	2.214440	2.262220	0.719975	0.059840
**clade IVb**	0.003520	0.003541	0.001423		2.242746	2.272880	0.689803	
**clade Va**	0.001414	0.001327	0.000636	0	0.599275	0.637773	0.660908	0.000028
**clade Vb**	0.001741	0.001681	0.000681		0.656810	0.661395	0.626301	

Table S20. Summary of the GO enrichment analysis of candidate genes.

GO ID	GO Term	GO Class	P-value	Adjusted P-value	Gene number
**GO:0048523**	negative regulation of cellular process	BP	0.000507	0.018275	2
**GO:0032515**	negative regulation of phosphoprotein phosphatase activity	BP	0.001605	0.018275	1
**GO:0042256**	mature ribosome assembly	BP	0.003207	0.031305	1
**GO:0050896**	response to stimulus	BP	0.004715	0.032845	5
**GO:0000077**	DNA damage checkpoint	BP	0.004807	0.032845	1
**GO:0004865**	protein serine/threonine phosphatase inhibitor activity	MF	0.001605	0.018275	1
**GO:0016667**	oxidoreductase activity, acting on a sulfur group of donors	MF	0.003719	0.032845	2
**GO:0008113**	peptide-methionine (S)-S-oxide reductase activity	MF	0.006404	0.042349	1
**GO:0043022**	ribosome binding	MF	0.007999	0.049689	1
**GO:0030896**	checkpoint clamp complex	CC	0.003207	0.031305	1

Table S21. Fst values between clades of *D. fruticosa*.

pop1	pop2	parameters	mean	median	std
low altitude (clade I)	high altitude (Clade II-V)	50 kb window, 10 kb step size	0.0842	0.079876	0.029431
clade IVb	clade IVa	50 kb window, 10 kb step size	0.1789	0.163381	0.07466
clade Vb	clade Va	50 kb window, 10 kb step size	0.3399	0.338487	0.115135

Table S22. The annotation results of SNPs and indels.

	Category	Number of SNPs		Category	Number of indels
	Upstream	246,174		Upstream	786,868
Exon	Stop gain	4,225	Exon	Stop gain	29,510
	Stop loss	494		Stop loss	1741
	Synonymous	434,188		Frameshift	409,827
	Non-synonymous	361,280		Non-frameshift	137,440
	Intronic	693,839		Intronic	1,456,861
	Downstream	234,959		Downstream	720,799
	upstream/downstream	45,709		upstream/downstream	143,004
	splicing	1,414		splicing	14,362
	Intergenic	933,921		Intergenic	2,888,943
	Total	2,956,203		Total	6,589,355

Table S23. The total number of ROH in the individuals of five clades.

Clade	Total number of ROH
**Clade I**	1437
**Clade II**	25679
**Clade III**	60985
**Clade IV**	1063
**Clade V**	58839
